# Development and *in vivo* evaluation of novel humanized CD19 CAR-T cells for advanced B cell malignancies

**DOI:** 10.3389/fimmu.2026.1798748

**Published:** 2026-05-26

**Authors:** Clara de Oliveira Andrade, Marcus Rafael Lobo Bezerra, Isabel Garcia Sousa, Eduardo Mannarino Correia, Ana Julia Ferreira Lima, Luiza Abdo, Emmanuel Arthur Albuquerque Aragão, Ronny Petterson dos Santos Araujo, Daniel Pasqualino Pinheiro, Igor Cabral Studart, João Vitor Steimbach, Marco Antônio Pretti, Gilvan Pessoa Furtado, Marcos Roberto Lourenzoni, Marcelo Macedo Brigido, Andréa Queiroz Maranhão, Martín Hernán Bonamino

**Affiliations:** 1 Cell and Gene Therapy Program, Research Coordination, National Cancer Institute (INCA), Rio de Janeiro, Brazil; 2 Protein Engineering and Health Solutions Group (GEPeSS) – Oswaldo Cruz Foundation (Fiocruz) – Eusébio, Ceará, Brazil; 3 Molecular Immunology Group, Biology Institute, University of Brasilia, Brasília, Brazil; 4 Graduate Program in Biotechnology of Natural Resources (PPGBiotec) – Department of Fisheries Engineering – Federal University of Ceará – Fortaleza, Ceará, Brazil; 5 Pasteur Fiocruz Center on Immunology and Immunotherapy, Eusébio, Ceará, Brazil; 6 Vice-Presidency of Research and Biological Collections (VPPCB), Oswaldo Cruz Foundation (FIOCRUZ), Rio de Janeiro, Brazil

**Keywords:** antibody humanization, CAR-T cell therapy, CD19, exhaustion markers, non-viral gene delivery, sleeping beauty

## Abstract

**Background:**

Chimeric antigen receptor T-cell therapy targeting CD19 has revolutionized the treatment of B-cell malignancies; however, limitations related to immunogenicity, persistence, and manufacturing costs remain significant barriers to broader clinical application. Most approved CD19-directed CAR-T products rely on murine-derived single-chain variable fragments, which may elicit anti-mouse immune responses and compromise long-term efficacy.

**Methods:**

Here, we report the design, characterization, and preclinical validation of two novel humanized anti-CD19 CAR constructs derived from the FMC63 antibody and generated using a non-viral Sleeping Beauty transposon system. Two humanized scFv variants, H1 and H2, sharing the same humanized light chain but distinct heavy chain frameworks, were evaluated for binding affinity, structural stability, and functional performance.

**Results:**

Although both humanized variants displayed reduced affinity relative to FMC63, they retained specific CD19 binding and supported robust CAR expression, activation, and memory differentiation in primary human T cells. *In vitro* cytotoxicity assays demonstrated comparable tumor cell killing and cytokine secretion across all constructs, including against CD19^low^ leukemia targets. *In vivo* xenograft models of standard and advanced B-cell acute lymphoblastic leukemia revealed that the H1 CAR-T cells achieved durable tumor control and overall survival comparable to FMC63, whereas the lower-affinity H2 construct showed reduced persistence and increased exhaustion marker expression.

**Conclusions:**

Collectively, these results demonstrate that rational humanization and harmonization of anti-CD19 scFvs can preserve antitumor efficacy, while mitigating functional exhaustion. This work supports the H1 construct as a promising candidate for further clinical development and highlights a scalable, cost-effective strategy for advancing locally manufactured CAR-T therapies in resource-limited settings.

## 1 Introduction

Cancer remains one of the leading causes of morbidity and mortality worldwide, with hematological malignancies such as B-cell acute lymphoblastic leukemia (B-ALL) posing significant therapeutic challenges, particularly in adult patients. Despite advances in chemotherapy, targeted therapies, and hematopoietic stem cell transplantation, patients with relapsed or refractory disease still face a poor prognosis. In this context, the landscape of oncology has been transformed by immunotherapy, now considered the “fifth pillar” of cancer treatment (1). Central to this shift is the development of Chimeric Antigen Receptor T-cell (CAR-T) therapy, which genetically modifies a patient’s T lymphocytes to express synthetic receptors targeting specific tumor antigens.

Adoptive T-cell therapies, particularly CAR-T therapy, have demonstrated remarkable clinical success in the treatment of relapsed/refractory B-cell malignancies (2). CAR-T cells are genetically engineered to express synthetic receptors that redirect T-cell specificity toward tumor related antigens, bypassing the limitations of HLA restriction and antigen presentation (3, 4). CD19-targeted CAR-T cell products, including those based on the murine-derived FMC63 single-chain variable fragment (scFv) (5), have been approved in several countries for the treatment of B-cell malignancies (6–8).

Despite the unprecedented clinical success of FDA-approved CAR-T products, significant challenges persist, including severe toxicities such as cytokine release syndrome (CRS) and immune effector cell-associated neurotoxicity syndrome (ICANS) (9), besides the high cost of viral-based manufacturing (10, 11). Although CAR-T has already demonstrated to be a game changer therapy, there is still room for improvement in every single aspect of the CAR-T design and production (12). Amongst these limitations and potential improvements, a primary concern is the immunogenicity of murine-derived single-chain variable fragments (scFvs), which may limit the persistence of CAR-T cells *in vivo* and preclude subsequent infusions due to the development of human anti-mouse antibody (HAMA) responses (13). Humanization of scFvs is a promising strategy to reduce these immune responses while preserving antigen specificity and functional activity. Previous preclinical studies have shown that humanized CAR constructs can reduce immune clearance, prolong CAR-T cell persistence, and maintain potent anti-tumor effects (14). These improvements are particularly relevant for patients requiring multiple infusions, as might be necessary for some hematological conditions and or solid tumor treatment (15). Humanization of scFvs, such as the FMC63, present in five out of six anti-CD19-based approved products, aims to reduce these effects, while maintaining high binding affinity.

Furthermore, the integration of non-viral gene delivery systems, such as the *Sleeping Beauty* transposon, offers a potentially safer and more cost-effective alternative to traditional viral vectors (12, 16). The use of this (and others) non-viral gene transfer systems represents one such innovation. This platform allows stable gene integration at reduced cost and complexity compared to viral vectors, offering a viable alternative for decentralized CAR-T manufacturing (17, 18). This approach is especially relevant in resource-limited settings, where the high cost of commercial CAR-T therapies remains a barrier to widespread clinical use. Beyond technological innovation, regional efforts have also emphasized the importance of preclinical testing platforms that reflect local infrastructure and patient needs (19). Developing robust, reproducible *in vitro* and *in vivo* models is essential for evaluating the efficacy and safety of new CAR constructs. These models contribute not only to scientific knowledge but also to the regulatory advancement and eventual clinical translation of CAR-T therapies in Latin America.

In this study, we present the functional characterization of two newly developed anti-CD19 CAR constructs incorporating humanized scFvs derived from the FMC63 antibody in the Sleeping Beauty system. Two humanized scFv versions (H1 and H2) harboring the same humanized VL, but different VH, derived from distinct human germlines, were designed. CAR-T cells were generated and evaluated by their phenotype, activation, and exhaustion profiles. Their cytotoxicity was assessed using both *in vitro* assays and *in vivo* xenograft models of B-ALL with varying levels of CD19 expression. Our data showed that the humanized versions were able to control tumor development and, in an affinity-dependent manner, improve overall survival, even for low-CD19-expressing tumor cells. The data presented here validate the innovative humanized scFv versions as candidates for further clinical trials to be held locally in Brazil.

## 2 Materials and methods

### 2.1 Cell lines

The human CD19+ B-cell ALL (acute lymphoblastic leukemia) cell line Nalm-6 was modified to express green fluorescent protein (GFP) and firefly luciferase (Luc) using lentiviral vectors and selected by cell sorting. The cells were maintained in a humidified incubator at 37 °C with a 5% CO_2_ atmosphere in RPMI-1640 medium (Gibco, CA) supplemented with 10% fetal bovine serum (FBS), 2 mM L-glutamine (Gibco), and 100 U/mL penicillin and 100 μg/mL streptomycin (pen/strep, Gibco). To generate CD19^Low^ Nalm-6, the gRNA sequence used for CRISPR-Cas9-mediated knockout was 5 ′CTGTGCTGCAGTGCCTCAA 3. Ribonucleoproteins (RNPs) were formed by incubating 90 pmol of gRNA with 30 pmol of Alt-R S.p. Cas9 Nuclease V3 (IDT, Newark, NJ, US) for 15 minutes at room temperature. Nalm-6 GFP+Luc+ cells (1 × 10^6^) were centrifuged at 100 g for 10 minutes at room temperature, resuspended in 100 μL of 1SM electroporation buffer (16), and transferred to a 0.2 cm cuvette (Mirus Biotech^®^, Madison, WI, US). Electroporation was performed using the X-001 pulse program on a Lonza^®^ Nucleofector^®^ IIb device (Lonza, Cambridge, MA, US). After electroporation, cells were resuspended in RPMI medium with 20% FBS without pen/strep and placed in a 24-well plate. Cells were sorted based on CD19-APC MFI expression using a MoFlo Astrios EQ (Beckman Coulter) (**Supplementary Figure 1**).

The human CD19+ B-cell Raji cell line (Burkitt’s lymphoma) and the human CD19- T-cell Jurkat cell line (Acute T-Cell Leukemia) were cultured in RPMI-1640 medium supplemented with 10% fetal bovine serum, 100 U/mL of penicillin, 100 μg/mL of streptomycin (pen/strep, Gibco) at 37 °C with a 5% CO_2_ atmosphere, in a humidified incubator.

### 2.2 Donors and PBMC preparation

Primary human cells were obtained from leukocyte reduction filters (RS-Haemonetics) provided by healthy donors from the INCA’s Blood Bank. The use of primary donor cells was approved by the Institutional Research Ethics Committee (CAAE: 42341021.7.1001.5274). All participants were informed of the study objectives and provided signed informed consent. Following negative serological screening for infectious diseases, leukocytes were recovered from the filters. Peripheral blood mononuclear cells (PBMCs) were eluted by back-flushing the filters with 80 mL of phosphate-buffered saline (PBS) and subsequently isolated via Ficoll-Hypaque-1077 density gradient centrifugation. Briefly, 40 mL of the diluted blood was carefully layered over 10 mL of Ficoll and centrifuged at 400 g for 25 minutes at room temperature. The resulting mononuclear cell interphase was collected and washed twice in PBS (400 g for 10 minutes). Finally, the cells were resuspended in PBS for quantification and viability assessment using the trypan blue exclusion assay.

### 2.3 Mice and xenograft model

For *in vivo* assays, immunodeficient mice NOD scid gamma-null (NSG), kept at INCA animal facility and acquired from Jackson Laboratories (Bar Harbor, Maine, US), were used. Female mice aged 8 to 12 weeks were randomized into control and experimental groups. Animal health was assessed three times per week until the onset of cachexia, after which daily monitoring was implemented. Welfare indicators - including posture, mobility, presence of diarrhea, and coat condition - were used to determine the humane endpoint for euthanasia, in strict accordance with CEUA (Ethics Committee on Animal Use) criteria. Euthanasia was performed via CO_2_ inhalation, in accordance with the AVMA Guidelines for the Euthanasia of Animals (2020) and the Brazilian CONCEA Normative Resolution RN 37/2018. Animals were placed in a euthanasia chamber exposed to 100% CO_2_ supplied from a commercial cylinder. CO_2_ was delivered using a pressure-reducing regulator and flowmeter to ensure a controlled displacement rate. The euthanasia chamber had internal dimensions of 28 cm (length) × 17 cm (width) × 13 cm (height), corresponding to a total volume of 6,188 cm³ (6.188 L). The CO_2_ flow was set to 2 L/min, which corresponds to 0.033 L/s. Based on the chamber volume, this represents a displacement rate of approximately 32.3% of the chamber volume per minute. Death was confirmed by the permanent cessation of the heartbeat via palpation. The chamber was cleared and cleaned between procedures to avoid residual odors.

### 2.4 Anti-CD19 constructs, cloning and expression

The amino acid sequences of FMC63 VH and VL were obtained from GenBank (MN702884). Humanization was performed by searching IgBlast for the closest human germinal V genes and grafting murine CDRs to those, as described earlier (20). The humanized sequences were also analyzed for their aggregation potential using Tango software (21). Based on this, a few amino acid changes were introduced into one of the humanized VHs (H1). Two versions (H1 and H2), harboring the same humanized VL, were constructed.

The humanized scFvs were cloned into two different vectors: pcDNA 3.4 for expression as soluble FvFc molecule (scFv fused to human IgG1 hinge-CH2CH3) (22) and pT4-19BBz Sleeping Beauty (23) for CAR production, harboring human CD8 spacer and transmembrane, 41BB intracellular and CD3 zeta chain activation domains. The framework of the 19BBz CAR was the one original construct described by Dario Campana`s group (24).

The soluble proteins were produced using the ExpiCHO™ Expression System (Thermo Fisher Scientific, Waltham, MA, USA) according to the manufacturer’s instructions. Briefly, the cells were transfected using ExpiFectamine CHO transfection reagent, and the cultures were grown in 125 mL flasks under agitation at 37°C in an 8% CO_2_ atmosphere in an Eppendorf incubator (S41i CO_2_ Incubator Shaker, New Brunswick, Edison, New Jersey, US). After 10 days of expression, culture supernatants were collected, and the recombinant proteins were affinity-purified using a Protein A column (Cytiva, Protein A HP, 1 ml, MA, USA), followed by size exclusion chromatography (SEC) (Cytiva, Superdex™ 200 Increase 10/300 GL, MA, USA) for polishing. The purity and yield for each construction were assessed by SDS-PAGE and quantified at 280 nm, adjusting for their respective extinction coefficients as determined by ProtParam analysis (Expasy). A western blotting using alkaline phosphatase-conjugated anti-Fc antibody was performed to confirm protein purification.

### 2.5 Cell binding of FvFc constructs

The purified proteins were tested by flow cytometry to confirm specific CD19+ cell binding. Briefly, 0.5 million cells (CD19+ Raji and CD19- Jurkat cell lines) were cultured and washed in FACS buffer (PBS, 1mM EDTA, 5% FBS). After washing, 5 or 50nM (in FACS buffer) of each FvFc were added for 30min at room temperature. An anti-Fc (Mouse anti-Human IgG1 Fc Secondary Antibody, AlexaFluor™ 488 (A10631), ThermoFisher, Waltham, MA, USA) was used for detection in a BD FACS Celesta^®^. Data were analyzed in FlowJo™ (v10.10).

### 2.6 Affinity measurement (BLI)

A recombinant version of extracellular CD19 (his-tagged, ACROBiosystems, Shanghai, China) was used as ligand in an Octet NTA Biosensor Kinetic Assay kit (Sartorius, Fremont, CA, US). Soluble versions (FvFc) of FMC63, H1, and H2 were used at the same concentrations as the analytes. All steps were performed as described by the manufacturers, at 30°C, 1000 rpm, in an Octet Red96 (Sartorius, Fremont, CA, US). Briefly, biosensors were equilibrated in 1X kinetics buffer (KB), then ligand capture (0.6 µg/mL his-tagged CD19) for 600 sec, followed by equilibration for 180 sec in KB. Association was carried out by dipping the biosensors into the respective KB-diluted analyte (FMC63, H1, or H2), at different concentrations, for 160 sec, followed by a dissociation step in KB for 300 sec. Soluble FvFcs were used in a concentration range from 37.5 nM down to 1.17 nM. As controls, one biosensor (no ligand/unloaded) was dipped into the highest analyte concentration and another biosensor was loaded with the His-tagged CD19 ligand and dipped into kinetics buffer (no analyte). No relevant non-specific binding was detected (<5% of analyte’s maximum concentration’s signal). Data were analyzed using Octet Analysis Studio 12.2.2.26, considering a 1:2 (bivalent analyte) model.

### 2.7 Molecular dynamic simulation

Molecular dynamics simulations were performed using homology models of FMC63 scFv (scFv_FMC63_) and its humanized variants (H1 and H2) named scFv_H1_ and scFv_H2_, constructed based on the FMC63–CD19 crystal structure (PDB ID: 7URV). The flexible (GGGGS)_3_ linker was modeled using Modeller version 10.4 (25), with model selection based on DOPE scores (26). The (scFv_FMC63_/CD19, scFv_H1_/CD19, and scFv_H2_/CD19) complexes were structurally aligned in PyMOL and simulated using GROMACS v2021.3 (27, 28), employing the CHARMM36 force field (29). Protonation states were assigned based on physiological pH (7.4) using the H++ server (30–32), and systems were solvated with TIP3P water (33). The complete atomic compositions of the systems are detailed in **Supplementary Table 1**. Bond constraints were applied using LINCS (34, 35), and SHAKE (36). Non-bonded interactions, including Van der Waals and electrostatic forces, were calculated using a 1.2 nm cutoff. Long-range electrostatic interactions were treated with the Particle Mesh Ewald (PME) method (37). Following energy minimization and NVT/NPT equilibration using V-rescale (38), while pressure was maintained at 1 atm (28, 39) during equilibration, and the Parrinello-Rahman barostat (40, 41) was used during the production run. The production phase consisted of 500 ns of MD simulation under NPT conditions, which was performed in triplicates (n1, n2, and n3), using the leap-frog integrator (42) with a time step of 2 fs. Trajectory snapshots were saved every 100 ps for subsequent analysis. Structural stability and binding energetics were evaluated by RMSD analysis and MM/PBSA calculations using gmx_MMPBSA (43). Detailed descriptions of the homology modeling procedures and molecular dynamics simulation setup are provided in the **Supplementary Methods**.

### 2.8 Peptide–HLA binding prediction

Peptides derived from FMC63, H1, and H2 scFv sequences were generated using a sliding window approach and evaluated for binding to human leukocyte antigen (HLA) molecules using NetMHCpan 4.2 and NetMHCIIpan 4.3. For MHC class I predictions, 9-mer peptides were assessed, while 15-mer peptides were used for MHC class II predictions. Predictions were performed across a panel of the most frequent HLA class I alleles (n = 12) and HLA-DRB1 alleles (n = 40) (list provided in **Supplementary Table 1**). Binding affinity was evaluated using %Rank scores, and peptide–HLA combinations with %Rank_EL < 2 for HLA-1, and %Rank_EL < 5 for HLA-2 were considered binders. All predictions were performed using default parameters. Downstream data processing, filtering, and visualization were conducted in R using the packages tidyverse and ggplot2 in a Rstudio environment.

### 2.9 CAR-T cell generation and expansion

For CAR-T cell generation, 3 x 10^7^ PBMCs were resuspended in 1SM buffer (44) containing 20 µg of plasmid pT4 (coding for CARs) and 1 µg of SB100X (tranposase harboring vector). Cells were electroporated with the Nucleofactor IIb system (Lonza, Basel, Switzerland) using 0.2 cm cuvettes (Mirus Biotech^®^, Madison, WI, USA) and program U-14. After electroporation, cells were transferred to a 6 well plate containing 2 mL of RPMI 1640 without antibiotics and supplemented with 20% SFB and IL-2 (50 U/mL). Two hours after electroporation, cells were incubated with 20 µL/well with anti-CD3/CD28 (T Cell TransAct, Miltenyi Biotec, Bergisch Gladbach, Germany). Cells were cultivated for 8 days, with reinoculation in RPMI 1640/10% SFB/IL-2 (50 U/mL) each 2 days. CAR expression was assessed by detecting CAR+ cells using a 9E10 anti-c-Myc FITC antibody (Alexa Fluor 647 or 488 conjugated, Santa Cruz, Dallas, TX, US), since CARs harbor a c-Myc tag. Activated CAR-T cells were used for phenotyping by *in vitro* cell toxicity assay, and *in vivo* anti-tumor experiments.

### 2.10 CAR-T cells phenotyping

For phenotypic and functional characterization of human T cells, multicolor flow cytometry panels were used, grouped into four categories: cytotoxicity/*in vivo*, activation, exhaustion, and memory. In the cytotoxicity and *in vivo* panel, CD3 was stained using clone UCHT1 conjugated to PerCP (BioLegend, San Diego, CA, US). c-Myc was detected with clone 9-E10 conjugated to Alexa Fluor 647 (Santa Cruz, Dallas, TX, US). Cell viability was assessed using the eFluor™ 780 viability dye (Thermo Fisher Scientific, Waltham, MA, US). For the activation panel, the following antibodies were used: CD4 (clone SK3, PerCP, BioLegend, San Diego, CA, US), CD8 (clone SK1, APC-Cy7, BioLegend, San Diego, CA, US), CD69 (clone FN50, PE, BioLegend, San Diego, CA, US), CD25 (clone PC61, Alexa Fluor 647, Invitrogen, Carlsbad, CA, US), and HLA-DR (clone LN3, PE, eBioscience, San Diego, CA, US). c-Myc expression was evaluated using clone 9-E10 conjugated to Alexa Fluor 488 (Santa Cruz, Dallas, TX, US). The exhaustion panel included CD4 (clone SK3, PerCP, BioLegend, San Diego, CA, US), CD8 (clone SK1, APC-Cy7, BioLegend, San Diego, CA, US), PD-1 (clone EH12.1, Alexa Fluor 647, Thermo Fisher Scientific, Waltham, MA, US), LAG-3 (clone 3DS223H, PE, BioLegend, San Diego, CA, US), and TIM-3 (clone F38-2E2, PE-Cy7, BioLegend, San Diego, CA, US). c-Myc was stained with clone 9-E10 conjugated to Alexa Fluor 488 (Santa Cruz, Dallas, TX, US). The memory panel consisted of CD4 (clone SK3, PerCP, BioLegend, San Diego, CA, US), CD8 (clone SK1, APC-Cy7, BioLegend, San Diego, CA, US), CCR7 (clone G043H7, PE, BioLegend, San Diego, CA, US), CD45RO (clone UCHL1, FITC, Thermo Fisher Scientific, Waltham, MA, US), and CD95 (clone DX2, PE-Cy7, BioLegend, San Diego, CA, USd). c-Myc detection was performed with clone 9-E10 conjugated to Alexa Fluor 647 (Santa Cruz, Dallas, TX, US). All analyses were performed according to the manufacturer’s instructions.

### 2.11 Cytotoxicity assay

T cells from three healthy donors were used to evaluate the cytotoxic potential of CAR-T cells engineered with the different constructs: FMC63, H1, and H2. GFP^+^ Nalm-6 tumor cells expressing either wild-type CD19 (CD19^WT^) or low CD19 levels (CD19^Low^) were used as target cells. The same number of target cells was used in all conditions, while the number of effector CAR-T cells varied. Donor-derived T lymphocytes were stained to determine the percentage of CAR-positive cells, and all conditions were adjusted to ensure equivalent CAR expression levels among constructs. Cells were plated at defined effector-to-target (E:T) ratios of 1:1, 0.5:1, 0.25:1, and 0.1:1, using a constant number of 1 × 10^5^ tumor cells per well. The cytotoxicity assay was conducted over a 96-hour period. GFP+ cells were determined at the initial incubation time and then every 24 hours. At each time point, cells were collected from the culture plates, stained with a Fixable Viability Dye eFluor 780 (Thermo Fisher Scientific, Waltham, MA, US), and analyzed by flow cytometry. Cytotoxic activity was assessed by quantifying the number of viable GFP-positive (GFP^+^) tumor cells. The decrease in GFP^+^ proportions over time was used as a readout for CAR-T-mediated cytotoxicity.

### 2.12 Cytokine quantification by ELISA

Enzyme-linked immunosorbent assay (ELISA) was used to quantify IL-2, TNF-α, and IFN-γ in supernatants collected from the cytotoxicity assay. Supernatants were obtained from one donor after 24 hours of co-culture and stored at −20°C until analysis. A volume of 100 µL from each sample was collected and later diluted 1:1 with RPMI medium. Cytokine concentrations were measured using DuoSet ELISA kits (R&D Systems, Minneapolis, MN, US), following the manufacturer’s instructions. Absorbance values were determined by absorbance at 540 nm. Concentrations were estimated by interpolation from a standard curve.

### 2.13 *In vivo* antitumor efficacy assay

Immunodeficient NOD scid gamma-null (NSG) female mice (8–12 weeks old) were housed at the INCA animal facility and randomly assigned to experimental groups. Two distinct *in vivo* antitumor efficacy assays were conducted to evaluate CAR-T performance against varying antigenic densities and disease states as previously described (45): a standard tumor model and an advanced disease model, utilizing Nalm-6 cell lines with differential CD19 expression levels. In both models, mice were injected intravenously (via the tail vein) with 1 × 10^5^ tumor cells in 100 µL PBS. Two Nalm-6 tumor cell lines were used: Nalm-6-GFP^+^Luc^+^CD19^WT^ and Nalm-6-GFP^+^Luc^+^CD19^Low^. Animals were inoculated via the tail vein with 100 µL of PBS containing 10^5^ cells of either Nalm-6-GFP-Luc-CD19^WT^ or Nalm-6-GFP-Luc-CD19^Low^ cell lines. Tumor burden was monitored weekly using bioluminescence imaging on an IVIS Lumina XR (Caliper Life Sciences, Inc., Hopkinton, MA, US). For this, mice received an intraperitoneal injection of 100 μL of D-luciferin (75 mg/kg), with signal acquisition occurring approximately 10 minutes post-injection. In the standard tumor model, 20 mice were used (n = 5 per group: vehicle, FMC63, H1, H2). Treatment was initiated 48 hours after tumor inoculation, with mice receiving 7 × 10^5^ CAR+ T cells in a total of 6.8 × 10^6^ T cells per mouse. In the advanced tumor model, 25 mice were used (n = 5 per group: Vehicle, Mock, FMC63, H1, H2). Treatment was initiated 11 days after tumor inoculation, with 1 × 10^6^ CAR+ T cells administered in a total of 7 × 10^6^ T cells per mouse.

### 2.14 Statistical analysis

Statistical analyses were performed using GraphPad Prism software (version 8.0, GraphPad Software, San Diego, CA, USA). For comparisons between two groups, a two-tailed paired or unpaired Student’s t-test was applied, as appropriate for the experimental design. For datasets involving three or more related groups, the nonparametric Friedman test was used. *Post-hoc* comparisons were conducted using Dunn’s multiple comparisons test for all-to-all group comparisons or for comparisons against a designated control group. For the survival curve, the Log-Rank test was used. Statistical significance was defined as p < 0.05 for all tests.

## 3 Results

### 3.1 Design of FMC63 humanized variants

**Figure 1:**
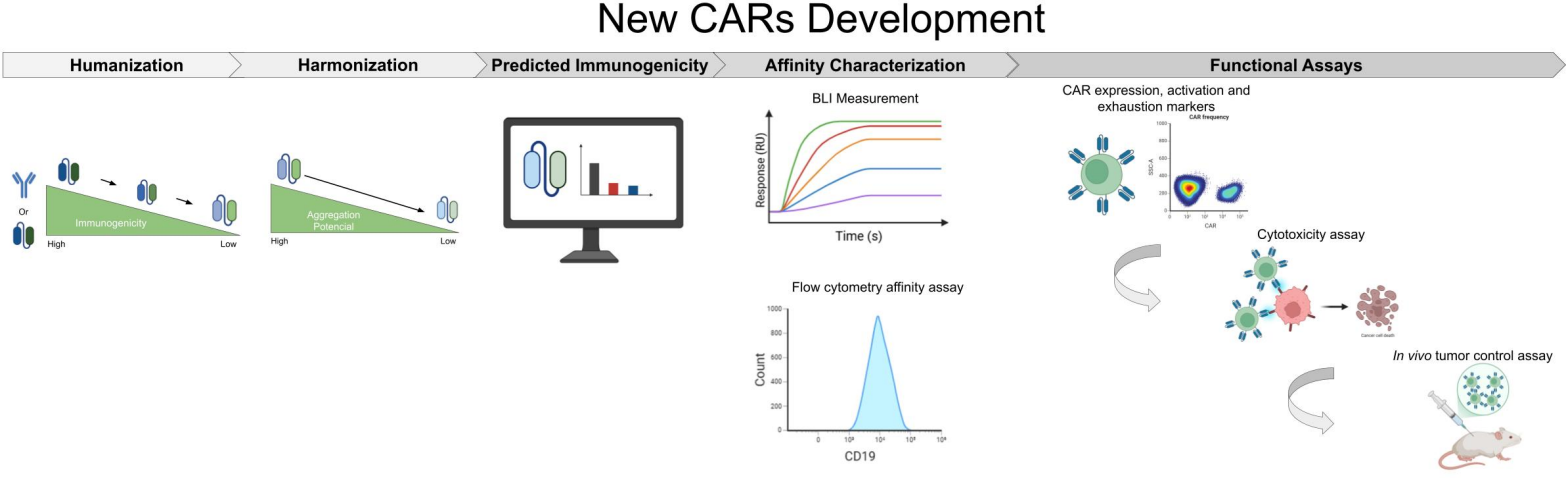
Integrated pipeline for design and validation of humanized and harmonized scFvs harboring CARs. The illustrated workflow begins with the humanization of murine variable domains from previously described CAR scFvs or other validated murine antibodies through CDR grafting onto human germline frameworks, followed by a harmonization step involving computational aggregation analysis. Potential candidates undergo immunogenicity screening via *in silico* HLA-binding predictions and are then produced as soluble proteins for precise characterization of affinity and antigen-recognition. The process concludes with *in vitro* functional validation of scFv-based CAR architectures via cytotoxicity and phenotypic assays, followed by *in vivo* therapeutic efficacy using NSG mouse models.

In this study a robust and reproducible workflow for the development of next-generation chimeric antigen receptors (CARs) was established by implementing an integrated pipeline focused on the precision engineering of single-chain variable fragments (scFvs) depicted in **Figure 1**. The process initiates with the humanization of murine variable domains via complementarity-determining region (CDR) grafting onto homologous human germline frameworks aiming to preserve binding paratopes, while reducing potential xenoreactivity. To address the inherent biophysical instabilities often associated with engineered fragments, the candidates are subjected to a computational analysis to identify potential aggregation-prone regions. This prompts a design refinement phase termed **“**harmonization**”,** involving targeted residues changes where necessary to improve stability without compromising affinity. Subsequently, the humanized or harmonized variants are screened for immunogenicity using predictive algorithms to identify potential HLA-binding epitopes, ensuring a reduced immunogenic profile for the new molecules. These new molecules are expressed as soluble counterparts to a more detailed characterization of their affinity and antigen recognition. These optimized scFvs are integrated into CAR architectures and subjected to comprehensive functional assays, including phenotypic profiling and cytotoxicity, to validate their therapeutic potential *in vitro*, followed by *in vivo* validation of therapeutic efficacy using NSG (NOD scid gamma) mouse models. This systematic approach not only facilitated the selection of the variants described herein but is also proposed as a standardized workflow for the streamlined design of clinical-grade biotherapeutics.

**Figure 2:**
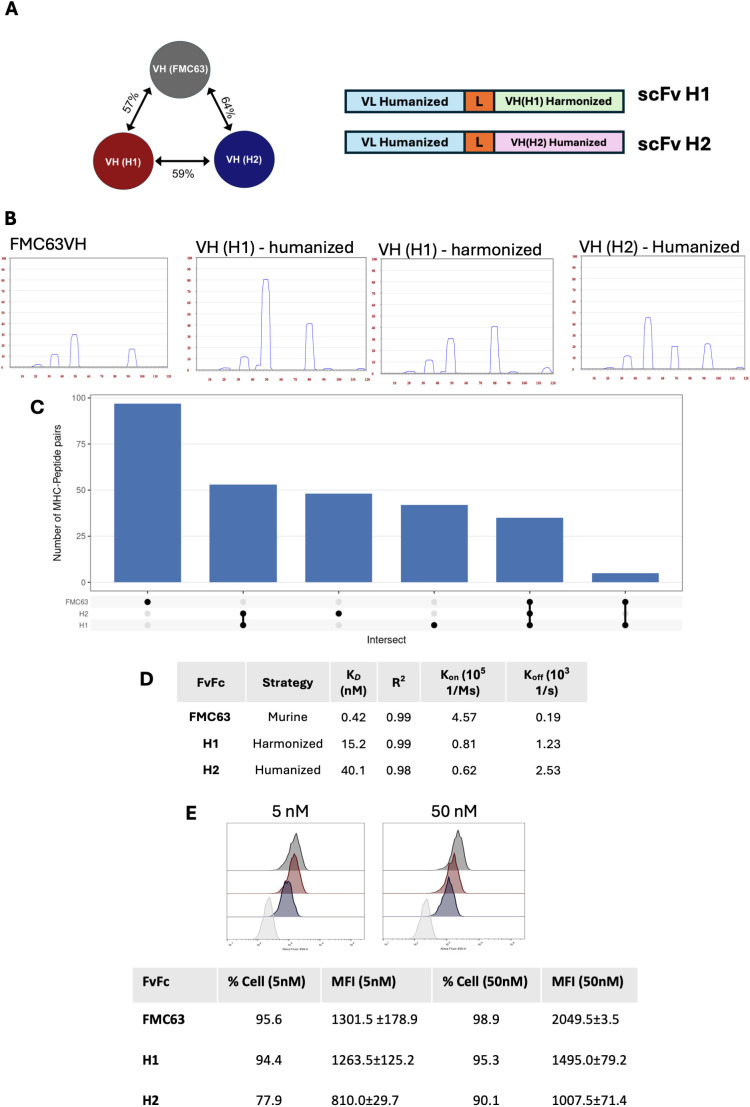
Design and characterization of humanized and harmonized anti-CD19 scFvs. **(A)** Sequence identity analysis comparing the FMC63 VH domain against the two most closely related human VH germlines (H1 and H2) utilized for humanization and harmonization. Schematic representation of the H1 (harmonized) and H2 (humanized) scFv constructs in a VL-linker-VH orientation. Both constructs utilize an identical humanized VL domain**. (B)** *In silico* prediction of aggregation propensity for FMC63, humanized and harmonized versions of H1 and humanized H2. **(C)** Prediction of HLA epitopes within scFvs sequences. Bar plots represent the number of predicted HLA-presented peptides for each unique or shared set among FMC63, H1, and H2, as indicated by the intersection matrix below. Filled dots denote the groups included in each intersection **(D)** Equilibrium dissociation constant (K_D_) of soluble FvFc fusion proteins (scFvs fused to a human IgG1 Fc region) as measured by biolayer interferometry (BLI). **(E)** Flow cytometric analysis of CD19+ Raji cells stained with the soluble FvFc molecules comparing antigen-binding properties.

Design of humanized scFvs was performed as described elsewhere (20). One version of the humanized VL domain was designed, while two different versions of VH, harboring distinct human germinal V genes, account for the two scFvs versions: H1 and H2, with FMC63 VH sequence identity of 57 and 64%, respectively (**Figure 2A**). The resulting H1 and H2 scFvs were constructed in a VL–linker–VH configuration, using a (GGGGS)_3_ linker (**Figure 2A**). To assess CAR aggregation propensity, we performed an *in silico* analysis using the TANGO algorithm (21) (**Figure 2B**). Initial analysis indicated that the H1 CDR grafted VH showed a high aggregation index, and few back mutations were introduced during its design, resulting in a “harmonized” (humanized and less aggregative) VH.

To evaluate the immunogenicity of the newly engineered scFv variants, we performed a comparative *in silico* analysis of predicted HLA epitopes between the parental murine FMC63 and the humanized H1 and H2 sequences (**Supplementary Figure 2**). This assessment utilized the most frequent MHC Class I and II alleles, as detailed in **Supplementary Table 2**. Our analysis revealed that humanization of the light chain variable domain (VL), which is shared by both variants, successfully reduced the density of predicted HLA-binding peptides within framework region 3 (FW3), although a similar reduction was not observed for FW2. The most significant decrease in potential HLA ligands was identified in the humanized and harmonized heavy chain (VH) peptides when compared to the original murine FMC63 VH. This improvement was particularly pronounced at the junction of CDR2 and FW3, as well as within the FW3 region itself. As summarized in **Figure 2C**, the comprehensive mapping of unique and shared epitopes confirms that the predicted immunogenic profile of the novel variants is substantially diminished relative to the parental antibody.

The corresponding genes were subsequently cloned into the pcDNA 4.3 expression vector to generate soluble FvFc fusion proteins. These FvFcs were used to determine binding affinity by biolayer interferometry (BLI) (**Supplementary Figure 3**), using the parental FMC63 scFv as a reference (K_D_ = 0.42 nM). Both humanized variants displayed a reduction in affinity, with H1 exhibiting a K_D_ of 15.19 nM and H2 a K_D_ of 40.1 nM (**Figure 2D**).

The soluble FvFc molecules were also used to assess antigen binding by flow cytometry on CD19+ Raji cells (**Figure 2E**). Despite their reduced affinities, both humanized FvFcs retained efficient CD19 binding, showing staining profiles comparable to that of FMC63 at saturating concentrations. However, in agreement with the BLI data, differences in binding efficiency were observed at a lower concentration (5 nM): FMC63, H1, and H2 stained 95.6%, 94.4%, and 77.9% of Raji cells, respectively. These differences became less pronounced at a higher concentration (50 nM), with 98.9%, 95.3%, and 90.1% of cells stained, respectively. Importantly, incubation of CD19− Jurkat cells with FvFcs at 50 nM resulted in fluorescence levels indistinguishable from the secondary antibody-only control, indicating the absence of detectable nonspecific binding (data not shown).

### 3.2 Structural stability and binding free energy analysis of scFv/CD19 complexes

The structural stability of the three scFv/CD19 complexes was evaluated by monitoring the root mean square deviation (RMSD) of Cα atoms over 500 ns of molecular dynamics (MD) simulations (**Supplementary Figures 4**-**S6**). In the present analysis, the flexible linker connecting the VL and VH chains was excluded, given its intrinsic mobility; the antibody fragment is therefore represented by the combined VL+VH domains. The modeled CD19 loops also exhibit high mobility. Therefore, the RMSD of CD19 was analyzed both including (CD19) and excluding these loops (CD19-Loops).

Overall, scFv_FMC63_ and scFv_H1_ displayed highly comparable stability profiles (**Supplementary Figures 4**, **S5**, respectively), characterized by rapid equilibration (stabilizing after ~50 ns), low RMSD values across VL and VH domains, and preserved global VL+VH conformations. CD19 remained stable in these systems, with mobility largely confined to loop regions. In contrast, the scFv_H2_ complex exhibited increased VH fluctuations, as reflected in higher global VL+VH RMSD values, while maintaining CD19 core stability, with enhanced flexibility localized to its loops (**Supplementary Figure 6**).

For the scFv_FMC63_/CD19 complex (**Supplementary Figures 7A–C**), the binding free energy remained consistently favorable across the three replicates, with mean values of –66.56 ± 10.68, –82.84 ± 10.46, and –70.65 ± 9.75 kcal·mol^−^¹ (**Table 1**), indicating a strong and stable association. The scFv_H1_/CD19 system (**Supplementary Figures 7D–F**) also exhibited favorable binding, with values of –70.72 ± 8.96, –59.07 ± 11.72, and –56.72 ± 8.75 kcal·mol^−^¹ (**Table 1**). Although replicates 2 and 3 presented slightly less favorable energies compared with scFv_FMC63_, replicate 1 showed a comparable affinity, suggesting that scFv_H1_ is capable of forming interactions of similar strength under certain conformational conditions (**Supplementary Figures 6D–F**). In contrast, the scFv_H2_/CD19 complex (**Supplementary Figures 7G–I**) displayed overall weaker binding, with binding free energy values of –50.36 ± 11.13, –57.27 ± 10.45, and –62.44 ± 10.32 kcal·mol^−^¹ (**Table 1**). Despite remaining energetically favorable, these values consistently indicate a reduced binding capability for scFv_H2_ relative to scFv_FMC63_ and scFv_H1_.

**Table 1:** Summary of the average ΔG_binding_ values obtained by MM/PBSA for the scFv/CD19 systems.

Systems	ΔG_binding_ (kcal.mol^-1^)		
	n1	n2	n3
scFv_FMC63_/CD19	-66.56 ± 10.68	-82.84 ± 10.46	-70.65 ± 9.75
scFv_H1_/CD19	-70.72 ± 8.96	-59.07 ± 11.72	-56.72 ± 8.75
scFv_H2_/CD19	-50.36 ± 11.13	-57.27 ± 10.45	-62.44 ± 10.32

Average ΔG_binding_ values were calculated, and the equilibrium time (teq) of 100 ns for all systems was considered. The averages and deviations presented correspond to the ΔG_binding_ profiles shown in **Supplementary Figure 6**.

### 3.3 Humanized scFv CAR-T cells maintain a phenotypic profile comparable to FMC63

**Figure 3:**
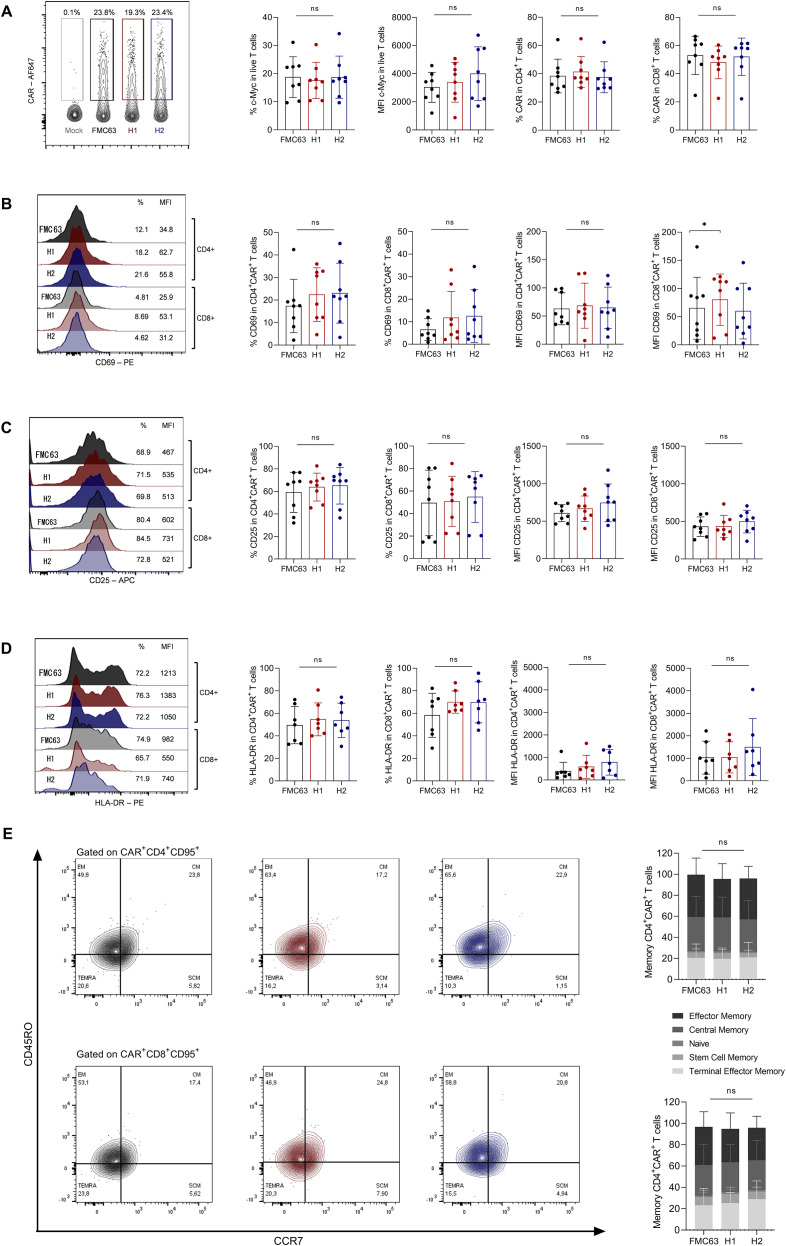
Analysis of CAR-T cell phenotypes. PBMC from eight different healthy donors were independently transfected with the three constructions. After 8 days of expansion and activation, cells were analyzed by flow cytometry. **(A)** Detection of CAR surface expression. CAR+ cells were identified using an anti-c-Myc antibody, targeting the N-terminal Myc-tag integrated into the CAR protein structure. **(B–D)** CAR+ Cells were also analyzed in terms of activation marker expression: CD69 **(B)**; CD25 **(C)** and HLA-DR **(D)**. Data are presented as both the percentage of positive cells and the Median Fluorescence Intensity (MFI) for both CD4^+^ and CD8^+^ cells. **(E)** The phenotype of CD4+ and CD8+ memory T cells was also performed, staining CAR+, CD25 + cells for CD45RO and CCR7 markers. The relative amounts of each: Central Memory (CM - CD95^+^, CCR7^+^ and CD45RO^+^), Stem Cell Memory (SCM - CD95^+^, CCR7^+^ and CD45RO^-^)), Terminal Differentiated (TEMRA - CD95^+^, CCR7^-^ and CD45RO^-^) and Effector Memory (EM - CD95^+^, CCR7^-^ and CD45RO^+^) cells are shown. Data were statically analyzed as described.

CAR constructs were cloned into the pT4 vector (23), flanked by Sleeping Beauty (SB) transposon repeat sequences. CAR proteins harbor one of the three scFvs (FMC63, H1 or H2), human CD8 spacer and transmembrane domains, human CD3 zeta chain and 41BB (CD137) costimulatory activation domains. PBMC from eight healthy donors were electroporated with 20 µg of pT4 vectors and 1 µg of SB100X (coding for sleeping beauty mutated for higher activity transposase). After 8 days of activation and expansion, the percentage, as well as the MFI of CAR+ cells varied among donors, but no significant difference was observed for the three constructs. An equivalent distribution was also observed between CD4+ and CD8+ CAR-T cells for the three constructs (**Figure 3A**). Notably, the CD8^+^subpopulation (means- FMC63: 53.03%; H1: 48.01%; H2: 52.03%) was larger than the CD4^+^ subpopulation (means - FMC63: 38.37%; H1: 41.25%; H2: 37.4%) across all constructs.

The activation profile of the expanded CAR-T cells was assessed by measuring the expression of CD69, CD25, and HLA-DR. With the exception of CD69 for CD8^+^ T cells harboring CAR H1, that showed a significant augmentation compared with FMC63 CAR-T cells (p=0.0373), no other significant differences in these markers were observed between the CD4^+^ and CD8^+^ populations for the three constructs (**Figures 3B–D**).

Furthermore, the memory differentiation profile of those expanded transfected T cell products was characterized using CD95, CCR7, and CD45RO staining (**Figure 3E**). T cell subsets were defined as follows: central memory (CD95^+^, CCR7^+^ and CD45RO^+^), stem memory cells (CD95^+^, CCR7^+^ and CD45RO^-^), terminal differentiated memory cells (CD95^+^, CCR7^-^ and CD45RO^-^), and effector memory cells (CD95^+^, CCR7^-^ and CD45RO^+^). Statistical analysis revealed no significant variation in the distribution of these memory subsets among the three CAR constructs for either CD4^+^ or CD8^+^ T cells. In all groups, the effector memory phenotype was the predominant population.

### 3.4 Long-term *in vitro* cytotoxicity of CAR-T cells

**Figure 4:**
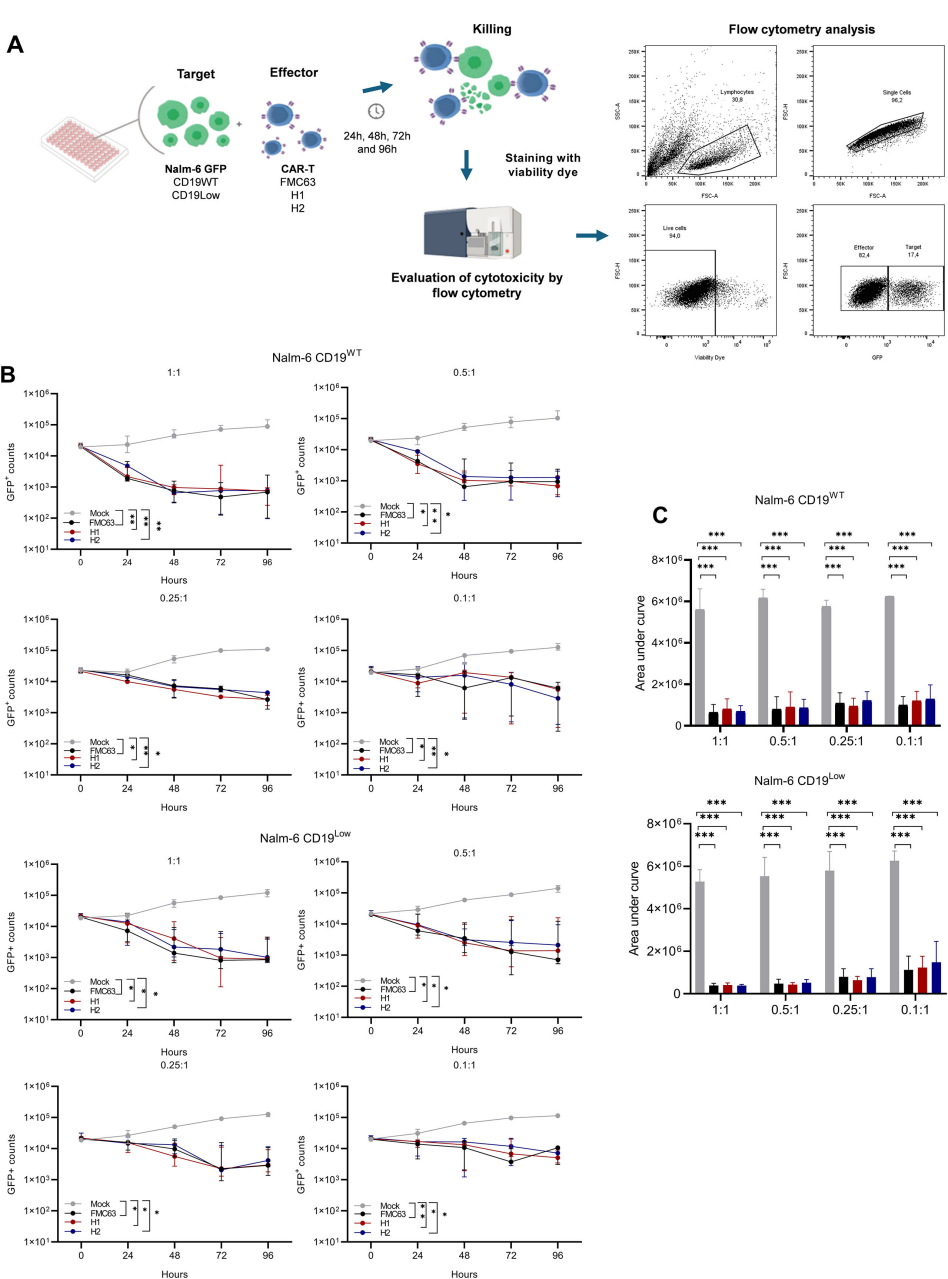
New anti-CD19 CAR-T cells maintain sustained cytotoxic activity during prolonged *in vitro* challenge. **(A)** Kinetic killing assays using GFP^+^ Nalm-6 target cells expressing either wild-type CD19 [CD19^WT^, **(B)** or reduced CD19 levels (CD19^Low^, **(C)**]. The count of residual GFP^+^ cells was quantified every 24 hours throughout a 96-hour co-culture period. Effector-to-target (E:T) ratios were assessed at 1:1, 0.5:1, 0.25:1, and 0.1:1. CAR+ cells (FMC63- black, H1- red and H2- blue). killing activity was compared to that exhibited by Mock-transduced negative control (grey). Cumulative cytotoxicity was compared using Area Under the Curve (AUC) analysis derived from the 96-hour killing kinetics. Assays were performed in triplicate and data are presented as mean ± SD. Statistical significance was determined via Two-Way ANOVA (*p < 0.05; **p < 0.01; ***p < 0.001).

The cytotoxic activity of expanded CAR-T cells was evaluated using long-term killing assays against Nalm-6 GFP^+^ leukemia cells. To assess CAR-T effector function against high or low target molecule expressing cells, target cells expressing either wild-type (WT) or low levels of CD19 were employed (**Figure 4A**). A fixed number of target cells was co-incubated with effector CAR-T cells at effector-to-target (E:T) ratios of 1:1, 0.5:1, 0.25:1, and 0.1:1. The kinetics of tumor cell elimination were monitored over 96 hours, with the absolute count of surviving GFP^+^ target cells quantified every 24 hours. Compared to Mock-transduced T cells, all CAR-T constructs demonstrated robust cytotoxic activity against both CD19^WT^ and CD19^Low^ targets (**Figure 4B**). As expected, cytotoxicity was most pronounced at higher E:T ratios (1:1 and 0.5:1) and against target cells with higher CD19 antigen density. Notably, Area Under the Curve (AUC) analysis revealed that the novel humanized constructs (H1 and H2) achieved cytotoxicity comparable to that of the parental FMC63 across both target cell types and at E:T ratios (**Figure 4C**).

To further characterize cytotoxic activity, cytokine secretion was quantified in supernatants after 24 hours of CAR-T and target cell co-culture (**Supplementary Figure 8**). The production levels of IL-2, TNF-α and IFN-γ were slightly diminished when CAR-T cells were challenged with CD19^Low^ targets, compared to CD19^WT^ cells. Critically, no significant differences in cytokine profiles were observed between the parental FMC63 and the humanized H1 and H2 constructs, being equivalent to the established FMC63 benchmark.

### 3.5 Phenotypic exhaustion marker kinetics in co-culture assays

To evaluate the impact of chronic antigen exposure on T cell fitness, the expression profiles of inhibitory receptors PD-1, TIM-3, and LAG-3 were longitudinally assessed prior to (0h) and following (96h) co-culture with CD19^WT^ Nalm 6 cells. Individual expression of those markers didn’t show any statistically significant difference among the distinct CAR-T cell groups in both co-culture time points (0 and 96h), either for CD4+ and CD8+ cells (**Supplementary Figure 9**). Despite this, all markers showed a tendency to increase their expression after 96 hours of co-culture. Except for H1 CD8^+^ CAR-T cells, as well for mock CD8^+^ cells, PD1 expression was higher for all other groups analyzed. Expression of LAG-3 also increased after 96hours for CAR+ co-cultures, being markedly high in H2 CD8^+^ CAR-T cells (72%). Prior to co-culture, TIM-3 was the least expressed marker across all groups. However, following the 96-hour co-culture, an increase in TIM-3 expression was observed in both H1 and H2 CD8+ CAR-T cells.

**Figure 5:**
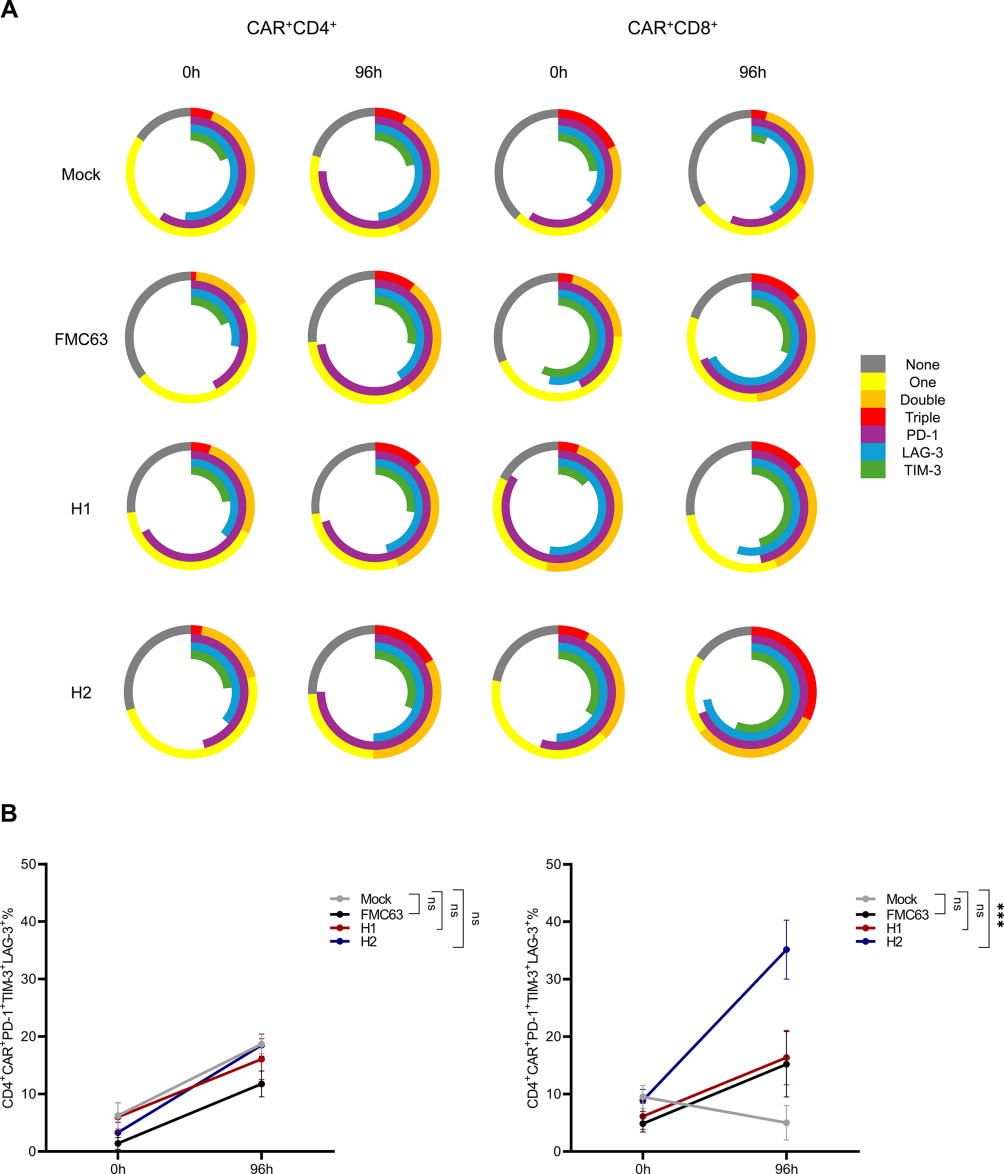
Exhaustion marker expression in novel CAR T cells post-antigen challenge. Expression of exhaustion markers were analyzed previously (0h) and after 96 h of co-culture with target cells. **(A)** Representative distribution of simultaneous expressions of: triple-positive (PD-1^+^LAG-3^+^TIM-3^+^; red), double-positive (any two markers; orange), and single-positive (PD-1^+^, purple; LAG-3^+^, blue; or TIM-3^+^, green). Negative cells for all markers are shown in gray. Data are stratified by CD4^+^ and CD8^+^ subsets. **(B)** Percentages of triple-positive effectors CD4+ and CD8 positive are shown at 0h and 96h post-antigen challenge. Mock controls are electroporated, non-transfected (CAR-) cells. Data represent the mean of three independent experiments; statistical analysis was performed using ANOVA. Data represent the mean of three independent experiments; statistical analysis was performed using Friedman test (***p< 0.001).

To further characterize the exhaustion landscape induced by chronic antigen challenge, a combinatorial analysis of marker expression was performed using the Boolean gating tool in FlowJo software. Following 96 hours of co-culture, the triple-positive (PD-1^+^LAG-3^+^TIM-3^+^) population increased across all effector groups, except for Mock CD8^+^ T cells, which decreased from 9.2% to 4.3% (**Figure 5A**). All CAR+ effector subsets exhibited an expansion of both double- and triple-positive populations. When comparing the CD4^+^ and CD8^+^ compartments, H2 CAR-T cells demonstrated the most pronounced induction of the triple-positive phenotype within the CD8^+^ subset, reaching 35%, after 96 hours of co-culture. In contrast, FMC63 and H1 CAR-T cells exhibited similar levels of triple marker expression (**Figure 5B**).

### 3.6 Pre-clinical validation of new CARs using two different tumor models

The tumoricidal activity of the novel humanized CARs was evaluated *in vivo* in immunodeficient NSG mice. Mice were xenografted with 1 x 10^5^ GFP^+^Luc^+^ Nalm-6 cell lines engineered with varying CD19 expression levels: CD19^WT^ and CD19^Low^ (**Supplementary Figure 1**), mimicking standard and advanced tumor stage, respectively.

**Figure 6:**
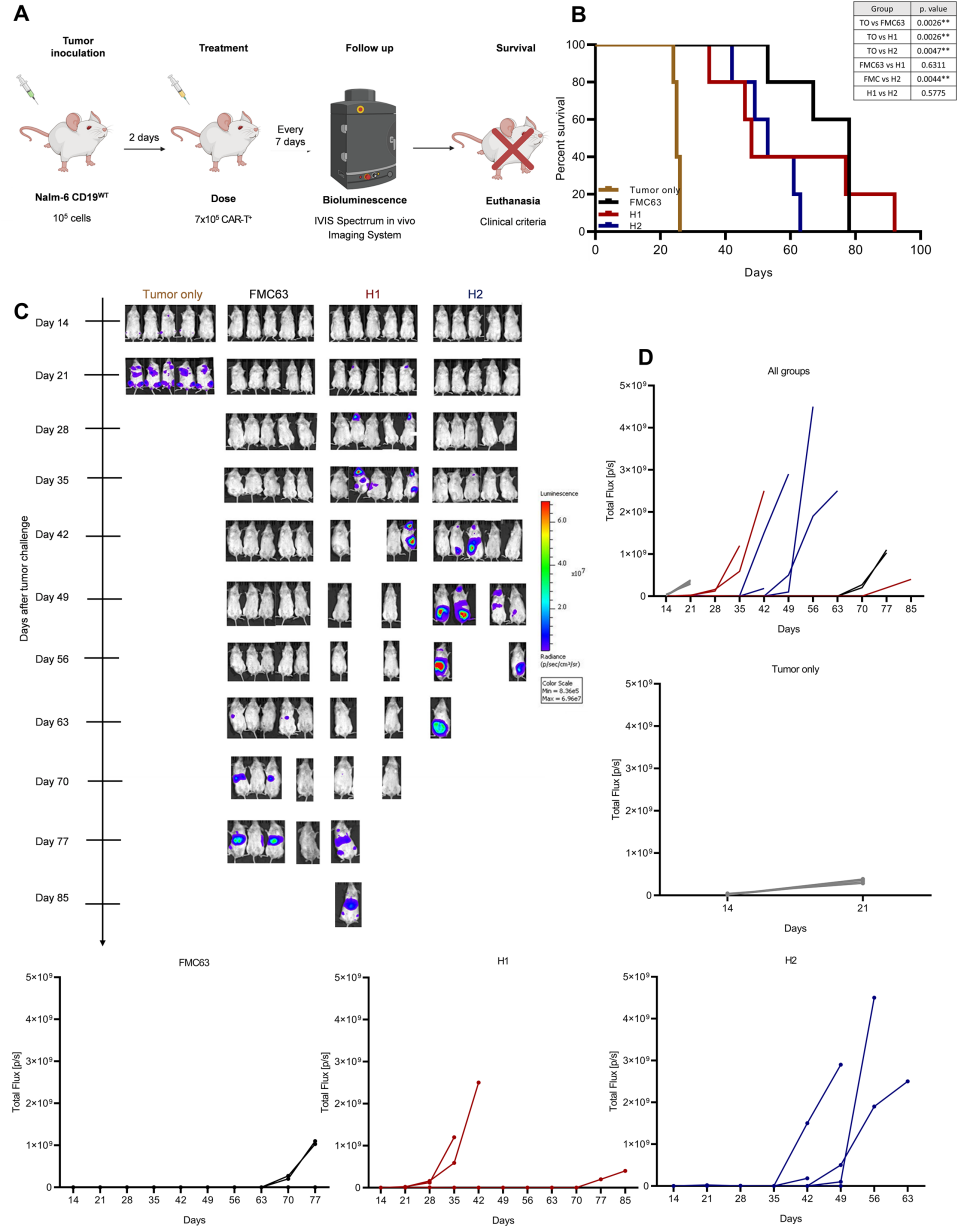
Comparative *in vivo* efficacy of CAR-T variants in a standard tumor burden xenograft model. **(A)** Schematic representation of the xenograft model used to evaluate CAR T-cell activity *in vivo*. NSG mice were inoculated with 1 × 10^5^ Nalm-6 CD19^WT^ cells. Two days after tumor inoculation, mice received a single intravenous dose of CAR-T cells (7 × 10^5^). Tumor burden was monitored by bioluminescence imaging using the IVIS Spectrum *in vivo* imaging system every 7 days. Mice were euthanized according to predefined clinical criteria, and survival was recorded. **(B)** Kaplan-Meier survival curves and statistical analysis of the advanced disease model. Experimental groups are indicated: Control Tumor Only (untreated mice, PBS-inoculated; brown) and CAR-T cells treated- FMC63 CAR-T (black), H1 CAR-T (red), and H2 CAR-T (blue). **(C)** Longitudinal assessment of tumor burden quantified by bioluminescence intensity (Total Flux, photons/second). **(D)** Kinetics of tumor progression and regression monitored by representative bioluminescence imaging across all experimental cohorts. End points were determined by ethical criteria for euthanasia.

Forty-eight hours after GFP^+^Luc^+^CD19^WT^ Nalm-6 inoculation, mice received a single intravenous dose of 7x10^5^ CAR+ cells/animal (**Figure 6A**). A control group (n=5) received PBS only. Overall survival is shown in **Figure 6B**. Tumor burden was assessed weekly by bioluminescence imaging, allowing visualization of disease localization (**Figure 6C**), and tumor progression was quantified as total photon flux (photons/s) (**Figure 6D**).

The control group reached ethical endpoints for euthanasia between day 24 and 26. The FMC63-treated group maintained tumor control until day 63. Although two of the five mice remained tumor-free until day 77, they were euthanized due to non-tumor related morbidity, including cachexia and hair loss most likely related to xenograft versus host disease due to the high amount of total human T cells infused (total dose of 6.8 x 10^6^ T cells). Animals treated with H2 CAR-T cells were able to control tumor growth until day 35, but no animals in this group survived beyond day 63. Two out of the five H1-treated animals showed tumor progression by day 21. Notably, two others remained tumor-free until day 70, with one of them exhibiting the longest overall survival among all treated animals.

Survival curves were analyzed using the Kaplan-Meier method (**Figure 6B**). All CAR-T treatment groups showed a significant increase in overall survival compared to the untreated control (p-value < 0.005). While all CAR groups showed a survival benefit, FMC63 and H1 treatments demonstrated comparable efficacy and significantly outperformed the H2 group.

**Figure 7:**
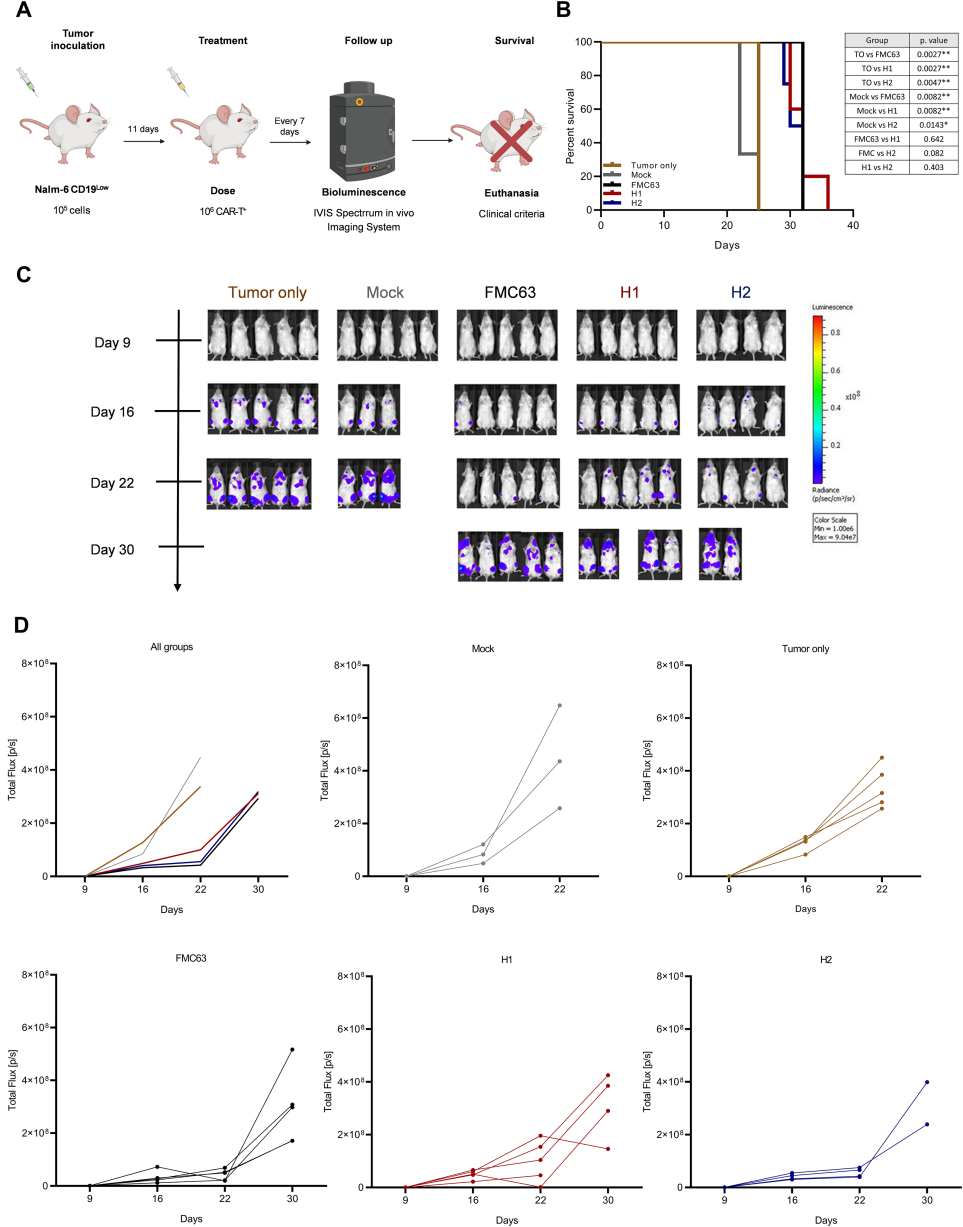
Antitumor activity of engineered CAR-T cells against established, advanced-stage tumors. **(A)** Schematic of the xenograft model used to evaluate CAR T-cell activity *in vivo*. NSG mice were inoculated with 1 × 10^5^ Nalm-6 CD19^Low^ cells. Eleven days after tumor inoculation, mice received a single intravenous dose of CAR T cells (1 × 10^6^). Tumor burden was monitored by bioluminescence imaging using the IVIS Spectrum *in vivo* imaging system every 7 days. Mice were euthanized according to predefined clinical criteria, and survival was recorded. **(B)** Kaplan-Meier survival curves and statistical analysis of the advanced disease model. Experimental groups are indicated as follows: vehicle control (brown), Mock (electroporated, non-transfected cells; gray), FMC63 CAR-T (black), H1 CAR-T (red), and H2 CAR-T (blue). **(C)** Longitudinal assessment of tumor burden and anatomical localization, quantified by bioluminescence intensity (Total Flux, photons/second). **(D)** Kinetics of tumor progression and regression monitored by representative bioluminescence imaging across all experimental cohorts.

The therapeutic efficacy of the novel CAR-T cells was further evaluated in an advanced tumor model. NSG mice were xenografted with 1 x 10^5^ GFP^+^Luc^+^CD19^Low^Nalm 6 (**Figure 7**). To simulate advanced-stage disease, CAR-T cell infusion (1 x 10^6^ CAR+ cells/animal within a total of 7 x 10^6^ T cells) was delayed until day 11 post-engraftment (**Figure 7A**). In this experiment, one animal in the H2 group died shortly after CAR-T cell infusion (in 24 hours) from undetermined causes. Although the exact cause could not be established, no clinical signs consistent with CAR-T–related toxicity were observed in the remaining animals. Possible contributing factors include those associated with handling and injection procedures.

By day 16, untreated and mock-treated mice exhibited significantly higher tumor burdens compared to all CAR-T treatment groups. At this time point, a single animal of FMC63 and H1 groups exhibited positive bioluminescence signals, which subsequently regressed by day 22 (**Figures 7B, C**). By day 22, the majority of control group mice (either untreated or mock) reached an ethical endpoint and were euthanized (**Figure 7B**). In contrast, FMC63 and H2 CAR-T treated mice showed low levels of tumor burden, being more homogenous among them than H1 CAR-T treated animals (total flux FMC63: 4,23e+007 p/s; H1: 1,4e+008 p/s; H2: 5,5725e+007 p/s) (**Figure 7D**). By day 30, disease progression was observed in all treatment cohorts. One of five mice in the H1 group and two of four in the H2 group had reached euthanasia criteria (**Figures 7B, C**). Notably, one animal in the H1 group showed a unique trend of tumor stabilization, with total flux decreasing from 1.96e+008 p/s at day 22 to 1.46e+008 p/s at day 30 (**Figure 7D**).

The overall survival (**Figure 7B**) varied from day 22 to 25 for untreated and mock groups, day 32 for FMC63-treated, day 30 to 32, with one animal reaching day 36 for the H1 group and day 29 to 32 for the H2 group, which was the least effective tumor control among the CAR groups. Statistical analysis (**Figure 7B**) confirmed that while no significant differences were found between the three CAR-T groups, all treatments significantly extended overall survival compared to untreated (p < 0.005) and mock-treated (p < 0.02) controls. Although some H1-treated animals were lost, this group consistently yielded individual survivors that outlasted the FMC63 cohort, leading to no significant statistical difference between the two treatments (**Figures 6B**, **7B**).

## 4 Discussion

The clinical landscape of B-cell malignancies has been transformed by CD19-directed CAR-T cell therapies. However, significant hurdles remain, including limited persistence, disease relapse in 30–50% of patients, and the immunogenicity of murine-derived scFvs (46, 47). Some efforts trying to generate less immunogenic CAR are being conducted leading to new receptors propositions harboring human (48) or humanized (49) scFvs. In this study, we constructed and functionally characterized two novel humanized CAR constructs, H1 and H2, developed through CDR-grafting. While H2 followed a conventional humanization protocol, H1 underwent structural “harmonization” designed to mitigate the protein aggregation often associated with framework substitution. Also, the humanization process reduces HLA epitopes, suggesting diminished immunogenicity.

Both humanized variants showed diminished affinity compared to their parental scFv FMC63, as measured by biolayer interferometry. This loss of affinity was corroborated by molecular dynamic simulations of the scFv-CD19 complex, in which the H2-CD19 complex was the most unstable. Notably, the H2 VH sequence is closer to the original FMC63 VH. Apparently, its framework had been more affected by the CDR graft than the more divergent H1 VH, leading to a less stable binding interface. Despite the differences in affinity, both humanized variants in their soluble forms (FvFc) were able to bind to CD19+ Raji cells, as observed by flow cytometry. Other authors who reported humanized antibodies based on FMC63, which used different combinations of germline V genes, had also developed effective CD19-based CAR-T cells (EP2970482A1, European Patent Office, 50, 51).

The reduced binding affinity observed in the humanized variants relative to the parental FMC63 did not compromise CAR-T cell functional efficacy. This suggests that the antigen-binding interface remains robust, corroborating the hypothesis that ligand interaction can mitigate structural constraints potentially introduced during the grafting of CDRs into a human framework (52). The relationship between CAR affinity and therapeutic efficacy remains a subject of significant debate. Affinity, as defined as a dissociation equilibrium, is not adequate to describe multiepitopic binding normally observed for membrane antigens, as suggested in a study with FMC63 (53). Therefore, the traditional “higher is better” paradigm should be taken cautiously. Moreover, while a high-affinity scFv ensures robust binding to the target antigen, it may also lead to excessive tonic signaling and rapid T cell exhaustion, particularly in high-tumor-burden environments. Some studies suggest that CARs with lower affinity, referred to as “low-affinity” or “fast-off-rate” variants, can achieve superior outcomes by allowing T cells to serially engage multiple target cells without becoming overstimulated (54).

The efficient killing of CAR T cells is highly dependent on antigen density and the formation of an immunological synapse (55, 56). In “antigen-low” scenarios, such as the CD19^Low^ model used in this study, higher affinity may be necessary to reach the signaling threshold required for T cell activation (56). These controversial findings suggest that there is no universal “ideal” affinity; rather, the efficacy of a CAR is the result of a delicate balance between binding strength, antigen density, and the resulting signaling kinetics that dictate the T cell’s metabolic and phenotypic fate. Of note, disease context might also impact the outcome. For instance, less persistent CAR-T cell products might be suitable for treating autoimmune diseases and as bridge therapy for leukemia, while persistent CAR-T cells might be necessary to keep aggressive tumors under check in the long term.

Our findings demonstrate that both humanized variants were comparable to FMC63 in terms of CAR and activation markers expressed (CD69, CD25, and HLA-DR). This finding in the resting state suggests that neither construct suffers from significant ligand-independent tonic signaling, a common pitfall in CAR design that can lead to premature exhaustion (57, 58). Furthermore, maintaining a memory-rich phenotype, characterized by high percentages of central memory (CM) and stem-cell memory (SCM) populations, is a promising indicator of long-term persistence, as these subsets are strongly correlated with superior clinical outcomes (59).

Long term cytotoxicity assay at very low effector to target rations failed to distinguish the efficacy of the humanized variants and FMC63. On the other hand, the standard-burden and advanced-burden (CD19^Low^) *in vivo* models provided a critical stress test that distinguished the two humanized candidates. While all CAR-treated groups significantly extended overall survival compared to controls, the H2 variant, which presented the lowest affinity, failed to sustain long-term tumor control for both models. This “early peak but rapid decline” profile likely correlates with our *in vitro* exhaustion data, in which H2-treated CD8+ cells exhibited a significant increase in the triple-positive inhibitory phenotype (PD-1^+^ TIM-3^+^ LAG-3^+^) following antigen challenge. The specific upregulation of TIM-3 in the H2 group can be associated with the reported loss of TCF1, a transcription factor essential for maintaining the stemness and proliferative capacity of T cells (60, 61). In contrast, the H1 variant, despite a more heterogeneous initial response in some mice, maintained a more favorable phenotypic profile and achieved longer overall survival, comparable to FMC63 in both models, suggesting a more balanced signaling kinetics. By avoiding terminal exhaustion, H1 can preserve a more robust pool of memory-like T cells, enabling the already observed serial killing and transient regression in advanced disease models (54). These results reinforce the notion that the success of a humanized scFv is not merely a function of binding strength (62), but of its ability to prevent the premature upregulation of inhibitory markers like LAG-3 and TIM-3. This aspect might be driven by the prevention of scFv aggregation and tonic signaling, although this hypothesis requests further investigation.

From a global health perspective, the development of stable, humanized CARs like H1 is a prerequisite for the next generation of affordable cellular therapies. In countries with universal health systems, such as Brazilian Unified Health System (SUS), the transition from expensive viral vectors to non-viral delivery platforms (e.g., transposon systems) is essential for sustainability. Accumulating evidence suggests that the *Sleeping Beauty* (SB) transposon system represents a viable and robust alternative for the validation of novel chimeric antigen receptor therapeutics (12). Following its initial clinical implementation for the generation of CAR-modified T cells, exemplified by studies conducted by research groups in the United States (17) and Germany (63) and the generation of cytokine-induced killer (CAR-CIK) cells by the Italian group (64), this non-viral gene delivery platform has consistently demonstrated a favorable clinical safety profile and promising therapeutic efficacy.

The clinical application of transposable elements for chimeric antigen receptor T-cell manufacturing has recently come under increased scrutiny following documented cases of therapy-related lymphoma in patients treated with *piggyBac* (PB)-modified cells (65) While the precise oncogenic drivers contributing to malignant transformation in these specific clinical cases remain to be fully elucidated, it is essential to consider the divergent mechanistic properties inherent to different transposon systems. Comparative studies indicate that *piggyBac* and *Sleeping Beauty* exhibit fundamental differences in their transpositional kinetics and genomic integration profiles, which may influence their respective safety outcomes (reviewed in 66). Despite these theoretical distinctions and the recent adverse report, these cases appear to be isolated. Currently, numerous clinical trials utilizing *piggyBac* are ongoing without further reports of such complications. Furthermore, longitudinal clinical experience with the *Sleeping Beauty* system—involving dozens of patients—has not yielded evidence of similar adverse events, suggesting a potentially distinct risk profile for this platform.

By pairing a high-fitness humanized scFv with decentralized manufacturing protocols, it is possible to significantly reduce the costs of CAR-T therapy, moving toward a model that is both biologically superior to murine-based products and financially accessible to universal public health frameworks like the Brazilian SUS. Future studies focusing on the immunological synapse and binding kinetics, as well as on novel VL combinations, can further elucidate the mechanisms by which H1 avoids the functional decline observed in H2 and may generate H1 variants with greater potential for clinical translation. Furthermore, the clinical implications of immunogenicity of these new variants and their impact on CAR-T cell persistence can only be definitively assessed in a clinical setting, although our predictions indicate that H1 and H2 should generate less immunogenic epitopes for most individuals.

In conclusion, we demonstrate that rationally humanized and harmonized anti-CD19 CAR constructs can achieve robust antitumor activity, while maintain favorable T-cell phenotypes, when delivered using a non-viral *Sleeping Beauty* platform. Among the two candidates evaluated, the H1 construct displayed a balanced affinity profile that translated into sustained *in vivo* tumor control, reduced exhaustion, and overall survival comparable to the parental FMC63 CAR, even in antigen-low disease settings. These findings underscore that CAR efficacy is governed not solely by binding affinity, but by the integration of structural stability, signaling kinetics, and T-cell fitness. Importantly, our work establishes a comprehensive preclinical pipeline for CAR design and validation that is compatible with decentralized, cost-effective manufacturing. This approach represents a critical step toward expanding access to CAR-T cell therapies and supports the clinical translation of humanized CARs tailored to diverse disease contexts and global health needs, representing a step forward in our local initiative for Brazilian CAR-T therapies. This pipeline will now be used for new targets.

## Data Availability

The original contributions presented in the study are included in the article/**Supplementary Material**. Further inquiries can be directed to the corresponding author.
