# Supplementary material for: Development and in vivo evaluation of novel humanized CD19 CAR-T cells for advanced B cell malignancies
Source: Front Immunol. 2026 May 26;17:1798748. doi: 10.3389/fimmu.2026.1798748 (PMC13247543; doi:10.3389/fimmu.2026.1798748)
Supplement: Supplementary file 1 [file DataSheet1.pdf]

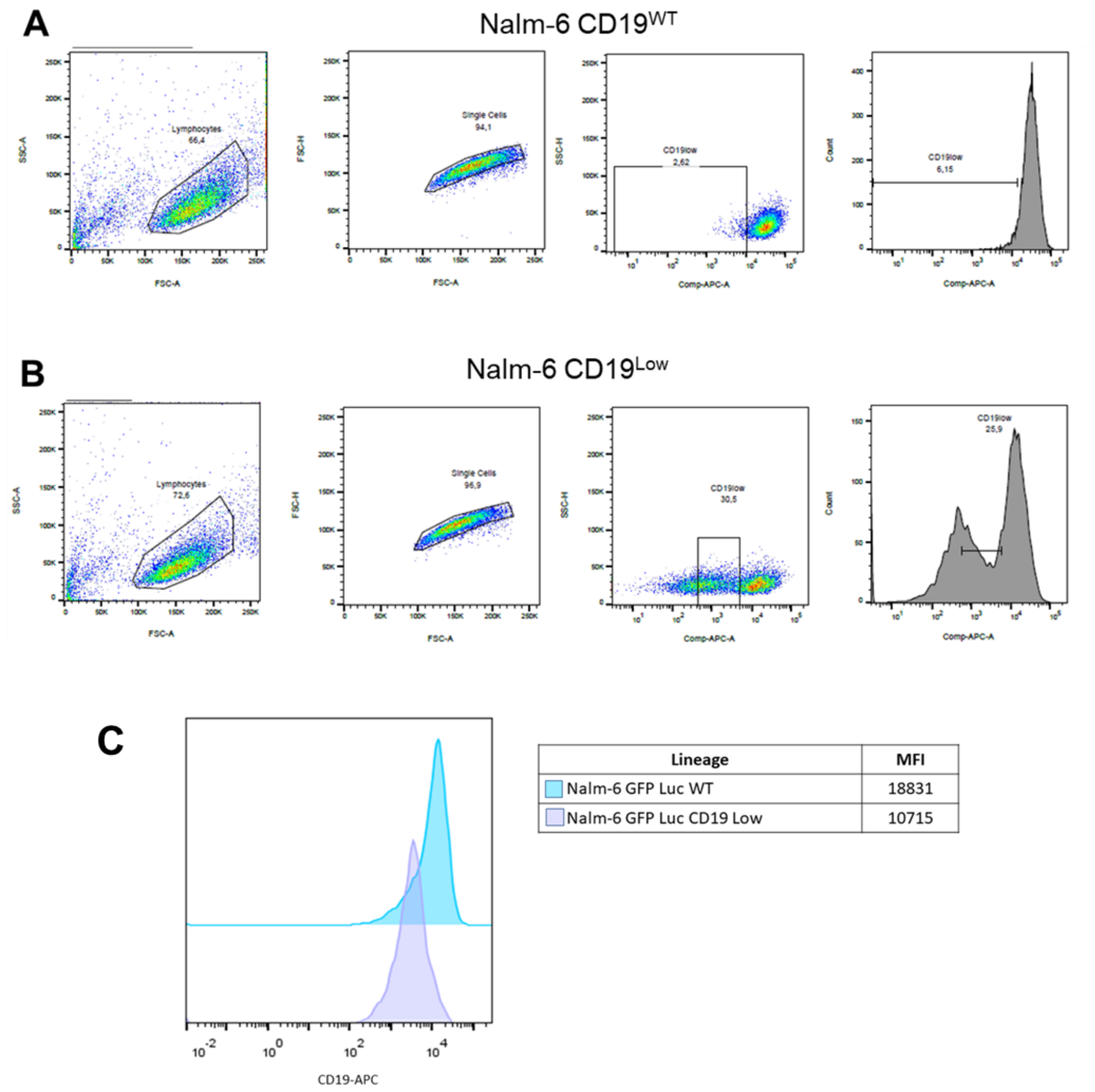

Supplementary Figure S1. Characterization and enrichment of CRISPR-edited CD19^{low} Nalm-6 cells. Assessment of CD19 expression levels via flow cytometry in (A) wild-type (WT) Nalm-6 cells and (B) CRISPR-edited Nalm-6 cells. (C) Post-sorting analysis comparing the enriched CD19^{low} population against the CD19^{WT} cell line.

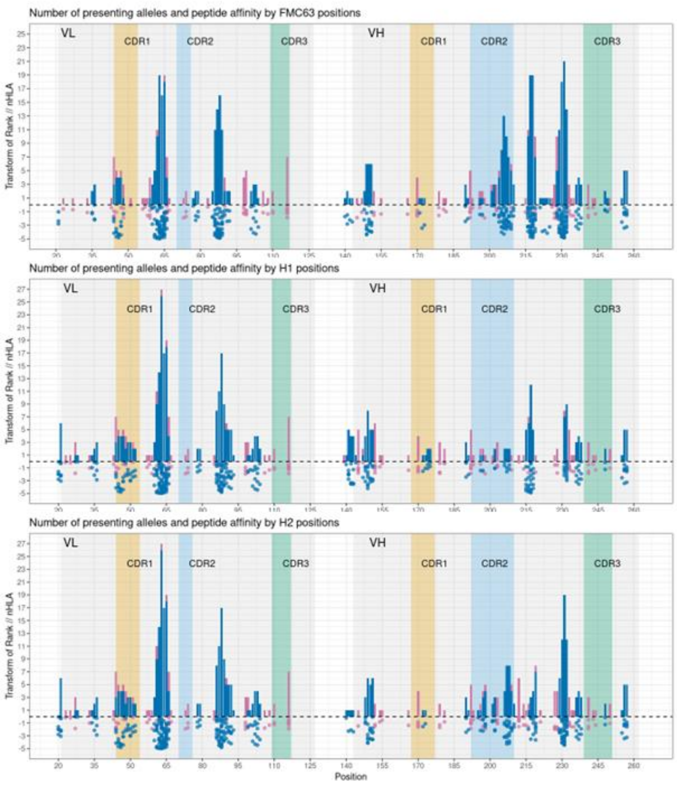

Supplementary Figure S2. Prediction of potential HLA binding peptides along scFv sequences. The number of presenting HLA class I (pink) and HLA-DRB1 (blue) alleles is shown as bar plots (positive y-axis), mapped according to peptide position along the scFv sequences of FMC63, H1, and H2. Shaded regions indicate complementarity-determining regions (CDRs) within the variable light (VL) and heavy (VH) chains. On the negative y-axis, individual points represent the transformed %Rank_EL values for each peptide–HLA interaction, where lower values indicate higher predicted binding affinity.

Supplementary Table S1
HLA Alleles Used to Find Potential Epitopes within scFv Sequences

Number	HLA_II	HLA_I
1	DRB1_0101	HLA-A01:01
2	DRB1_0102	HLA-A02:01
3	DRB1_0301	HLA-A03:01
4	DRB1_0302	HLA-A24:02
5	DRB1_0401	HLA-A26:01
6	DRB1_0402	HLA-B07:02
7	DRB1_0403	HLA-B08:01
8	DRB1_0404	HLA-B15:01
9	DRB1_0405	HLA-B27:05
10	DRB1_0407	HLA-B39:01
11	DRB1_0411	HLA-B40:01
12	DRB1_0412	HLA-B58:01
13	DRB1_04140	NA
14	DRB1_04155	NA
15	DRB1_0701	NA
16	DRB1_0704	NA
17	DRB1_0802	NA
18	DRB1_0803	NA
19	DRB1_0804	NA
20	DRB1_0901	NA
21	DRB1_1001	NA
22	DRB1_1101	NA
23	DRB1_1102	NA
24	DRB1_1209	NA
25	DRB1_1104	NA
26	DRB1_1201	NA
27	DRB1_1301	NA
28	DRB1_1202	NA
29	DRB1_1302	NA
30	DRB1_1303	NA
31	DRB1_1304	NA
32	DRB1_1401	NA
33	DRB1_1402	NA
34	DRB1_1404	NA
35	DRB1_1408	NA
36	DRB1_1409	NA
37	DRB1_1501	NA
38	DRB1_1502	NA
39	DRB1_1503	NA
40	DRB1_1602	NA

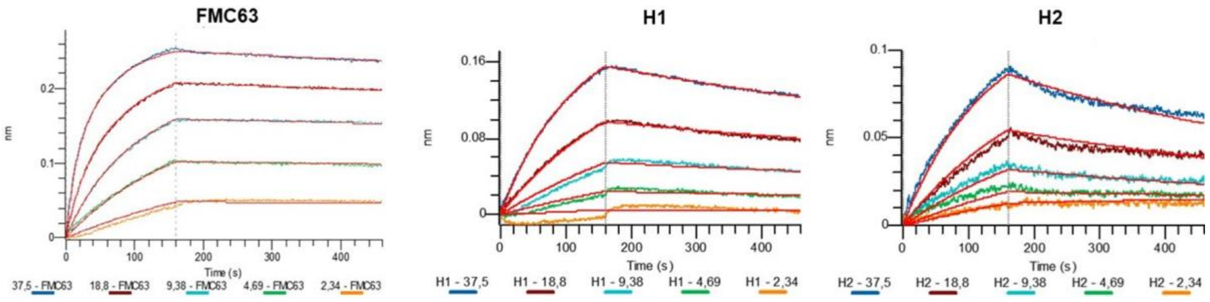

Supplementary Figure S3. **Biolayer interferometry (BLI) analysis of the binding kinetics of murine (FMC63) and humanized (H1 and H2) anti-CD19 FvFc soluble proteins**. Recombinant His-tagged extracellular CD19 was immobilized on Ni-NTA biosensors at 0.6 µg/mL. Murine FMC63 and the humanized variants H1 and H2 were analyzed over a serial dilution ranging from 37.5 nM to 2.34 nM, as indicated by colors below the graphs. Association and dissociation phases are shown for each analyte concentration. Sensorgrams used for equilibrium dissociation constant (K_D) determination were fitted using a 1:2 bivalent analyte binding model (red fitted curves). The estimated K_D values for murine FMC63, H1, and H2 were 0.42 nM, 15.2 nM, and 40.1 nM, respectively.

Supplementary Methods

Molecular Dynamics Simulations

For molecular dynamic simulation, three-dimensional structures of scFvs from FMC63 and its humanized versions were assembled based on the crystal structure of FMC63 available in the Protein Data Bank (PDB ID: 7URV), named here as $scFv_{FMC63}$. The flexible $(GGGGS)_3$ linker was modeled using Modeller version 10.4. A total of 250 homology models were generated, and the one showing the best DOPE score was selected. The same strategy was applied to generate homology models of two humanized scFv variants, named $scFv_{H1}$ and $scFv_{H2}$, using the complete $scFv_{FMC63}$ structure as a template. CD19 three dimensional structure was also obtained from PDB ID: 7URV. Additional homology modeling was carried out using Modeller version 10.4 to resolve some loop regions missed in PDB data.

Modeled scFvs and CD19 structures were aligned to the FMC63-CD19 crystallographic template using PyMOL (v3.1.3, Schrödinger, LLC). The resulting complexes ($scFv_{FMC63}$/CD19, $scFv_{H1}$/CD19, and $scFv_{H2}$/CD19) maintained the native binding interface. Molecular dynamics simulations were carried out using GROMACS version 2021.3 (27,28), employing the CHARMM36 force field. Protonation states were assigned based on physiological pH (7.4) using the H++ server. The complexes were solvated in a dodecahedral simulation box filled with TIP3P water molecules, ensuring a minimum distance of 1.8 nm between the protein and the box boundaries in all directions. The complete atomic compositions of the systems are detailed in Supplementary Table 1. Protein bond constraints were applied using the LINCS algorithm, and the geometry of water molecules was preserved using the SHAKE method. Non-bonded interactions, including van der Waals and electrostatic forces, were calculated using a 1.2 nm cutoff. Long-range electrostatic interactions were treated with the Particle Mesh Ewald (PME) method.. Each system underwent energy minimization in two steps: first, with the steepest descent algorithm, followed by further refinement using the conjugate gradient method. To replicate physiological ionic strength (0.15M), Na^+ and Cl^- ions were added to neutralize the systems.

Equilibration was performed in both NVT and NPT ensembles. The NVT equilibration included two phases: an initial 7.5 ps stage with the scFv and CD19 atoms frozen, followed by a 20 ps stage in which only protein backbones were frozen. This was followed by 55 ps of NPT equilibration. Temperature was kept at 310 K using the V-rescale thermostat, while pressure

was maintained at 1 atm using the Berendsen barostat during equilibration, and the Parrinello-Rahman barostat during the production run. The production phase consisted of 500 ns of MD simulation under NPT conditions, which was performed in triplicates (n1, n2, and n3), using the leap-frog integrator with a time step of 2 fs. Trajectory snapshots were saved every 100 ps for subsequent analysis.

To evaluate the stability of the scFvs/CD19 complexes during the simulations, root-mean-square deviation (RMSD) analyses were performed using the GROMACS version 2021.3 software package. Structural deviations were assessed by calculating the RMSD of the Cα atoms using the *gmx rms* tool, with all frames aligned to the initial structure of the MD trajectory. Binding free energy (ΔG_{bind}) was calculated using gmx_MMPBSA (43), based on 2500 frames from the MD trajectories. The analysis used CHARMM force field parameters and the following input settings: ionic strength of 0.15 M, fill ratio of 4.0, and radiopt=0, which applies the atomic radii from the topology file for both polar and nonpolar solvation terms. All results were visualized and plotted using Origin 8.0.

Supplementary Table S2.
Composition of the simulated molecular dynamics systems.

Overview of system nomenclature, number of water molecules, and ions for each system. All systems comprise scFvs interacting with the CD19 target. The native FMC63-derived scFv system is represented as $scFv_{FMC63}$/CD19, while humanized variants are represented as $scFv_{H1}$/CD19 and $scFv_{H2}$/CD19.

System (n1, n2 and n3)	Number of water molecules	Na^+	Cl^-
$scFv_{FMC63}$/CD19	56117	164	162
$ScFv_{H1}$/CD19	62128	179	179
$scFv_{H2}$/CD19	65779	190	191

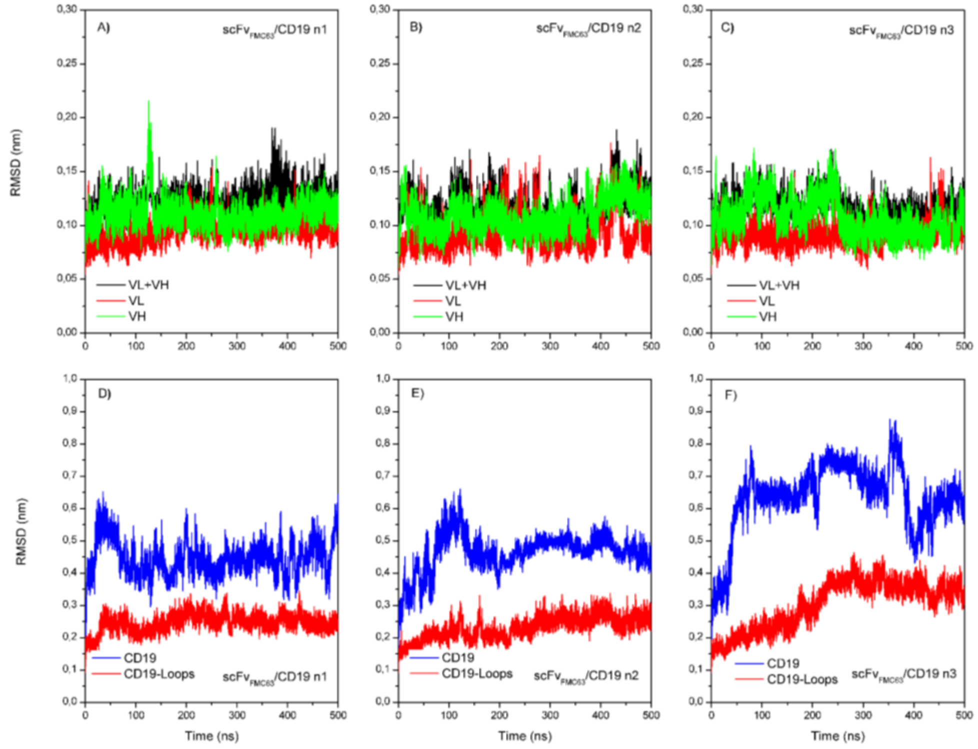

Supplementary Figure S4. RMSD profiles of the Cα atoms of VL+VH, VL, VH and CD19 structures for scFvFMC63/CD19 system over 500 ns of MD simulation. (A-C) RMSD profile of scFvFMC63 in triplicates n1, n2 and n3, respectively. (D-F) RMSD profile of CD19 in triplicates n1, n2 and n3, respectively. The structures obtained during the MD simulations were compared to their respective initial reference structures. The scFv linker was excluded from the RMSD analysis due to its high flexibility, being rich in glycine and serine residues, which results in large fluctuations. The scFv excluding the linker is referred to as VL+VH. The CD19 structure excluding the modeled loops is denoted as CD19-Loops.

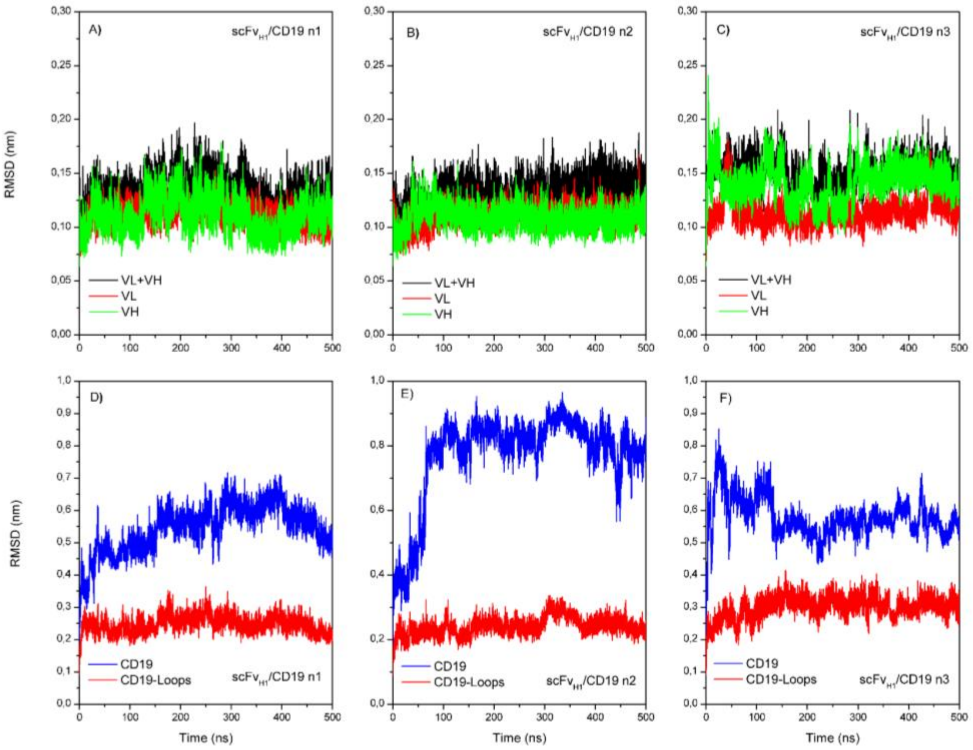

Supplementary Figure S5. RMSD profiles of the Cα atoms of VL+VH, VL, VH and CD19 structures for scFv$_{H1}$/CD19 system over 500 ns of MD simulation. (A-C) RMSD profile of scFv$_{H1}$ in triplicates n1, n2 and n3, respectively. (D-F) RMSD profile of CD19 in triplicates n1, n2 and n3, respectively. The structures obtained during the MD simulations were compared to their respective initial reference structures. The scFv linker was excluded from the RMSD analysis due to its high flexibility, being rich in glycine and serine residues, which results in large fluctuations. The scFv excluding the linker is referred to as VL+VH. The CD19 structure excluding the modeled loops is denoted as CD19-Loops.

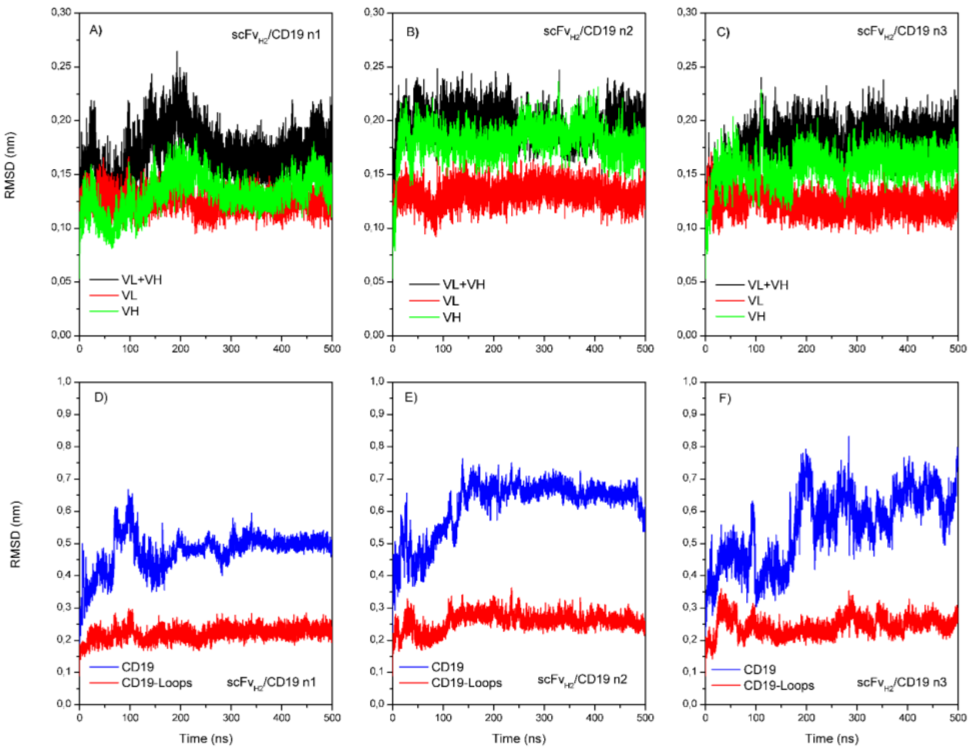

Supplementary Figure S6. RMSD profiles of the Cα atoms of VL+VH, VL, VH and CD19 structures for $scFv_{H2}$/CD19 system over 500 ns of MD simulation. (A-C) RMSD profile of $scFv_{H2}$ in triplicates n1, n2 and n3, respectively. (D-F) RMSD profile of CD19 in triplicates n1, n2 and n3, respectively. The structures obtained during the MD simulations were compared to their respective initial reference structures. The scFv linker was excluded from the RMSD analysis due to its high flexibility, being rich in glycine and serine residues, which results in large fluctuations. The scFv excluding the linker is referred to as VL+VH. The CD19 structure excluding the modeled loops is denoted as CD19-Loops.

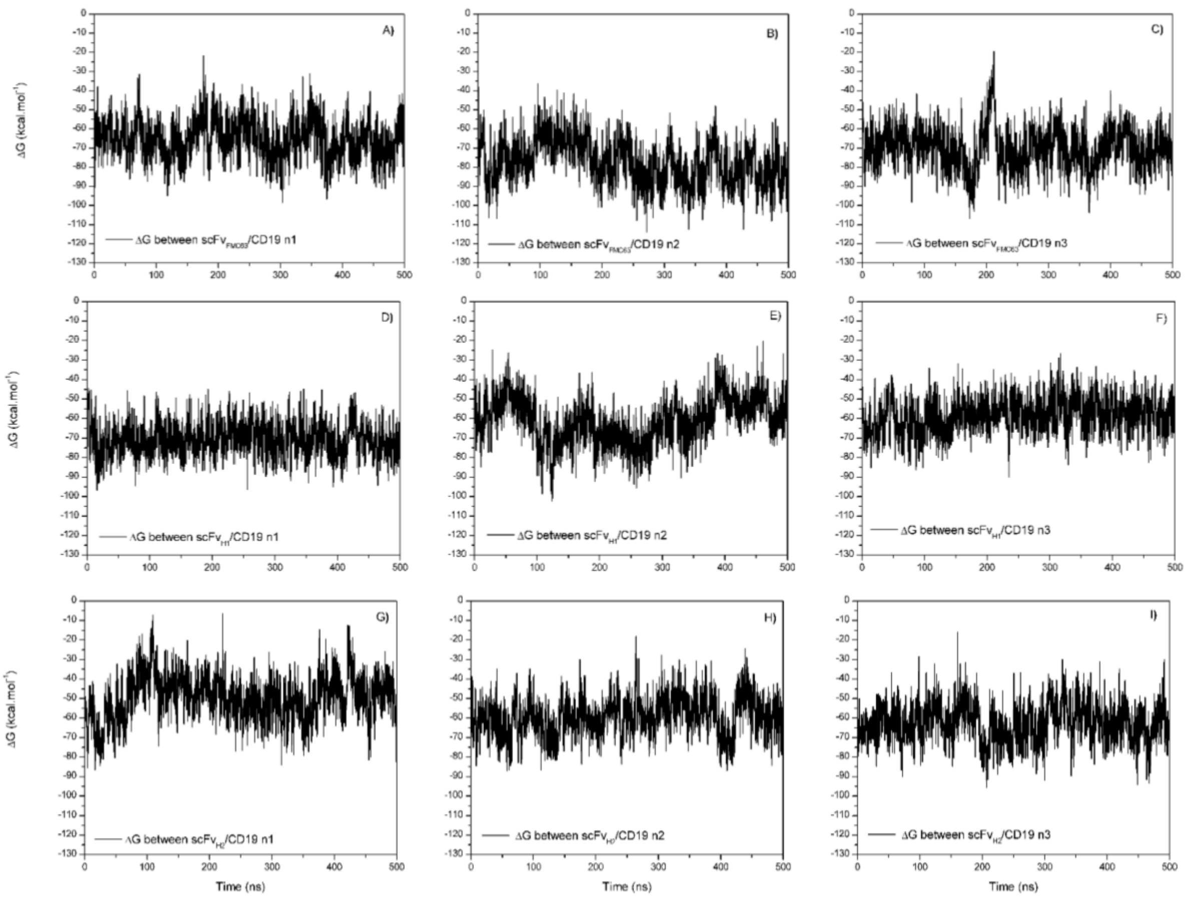

Supplementary Figure S7. $\Delta G_{Binding}$ curves of the complexes scFvs/CD19 over the simulation. (A-C) $scFv_{FMC63}$/CD19 system. (D-F) $scFv_{H1}$/CD19 system. (G-I) $scFv_{H2}$/CD19 system.

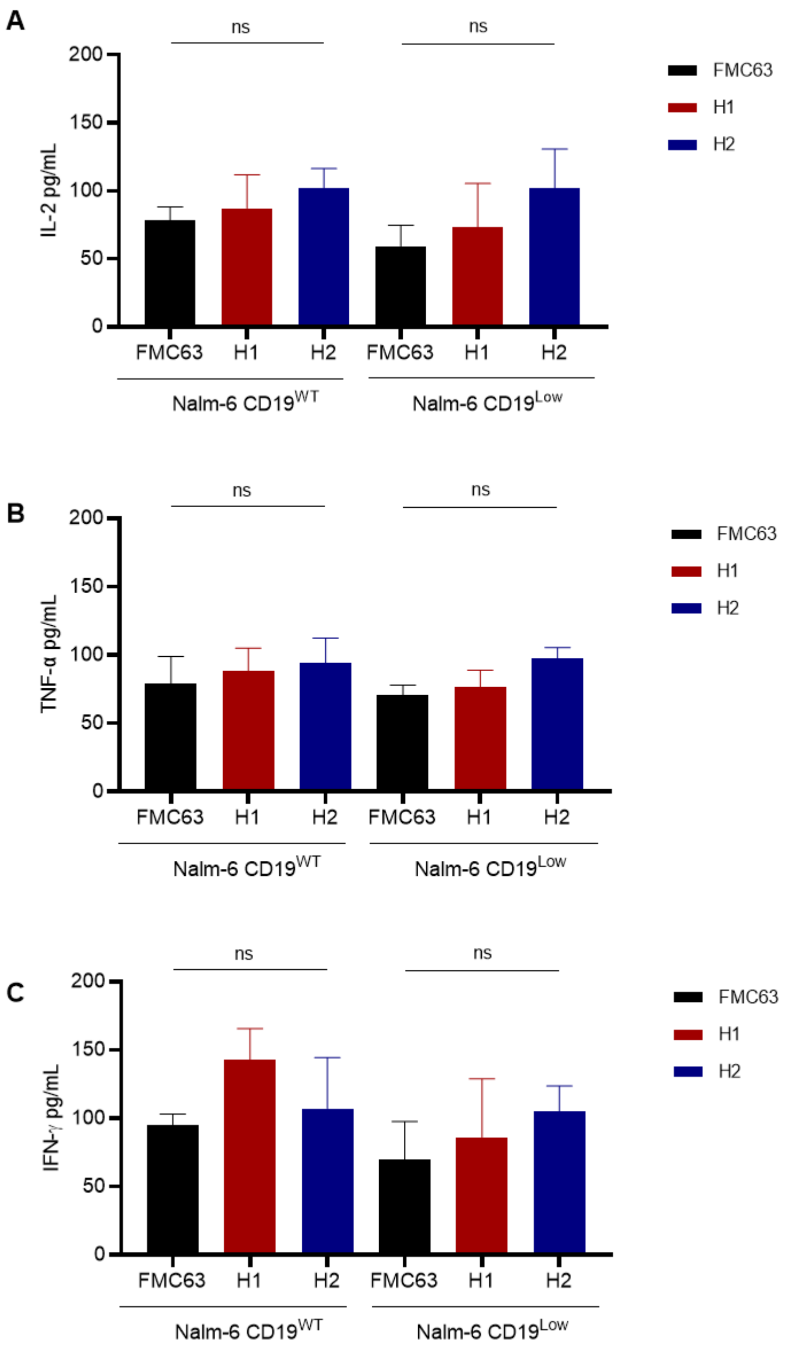

Supplementary Figure S8. Cytokine secretion profile of humanized CAR-T cells. Cytokine concentrations were quantified in the supernatants of CAR-T cells co-cultured with either Nalm-6 CD19^{wt} or Nalm-6 CD19^{low} target cells for 24 hours. (A) IL-2 levels, (B) TNF-α levels, and (C) IFN-γ levels. The experimental groups are represented by color: FMC63 (black), H1 (red), and H2 (blue). Data represent a single representative independent experiment; measures were performed in triplicates.

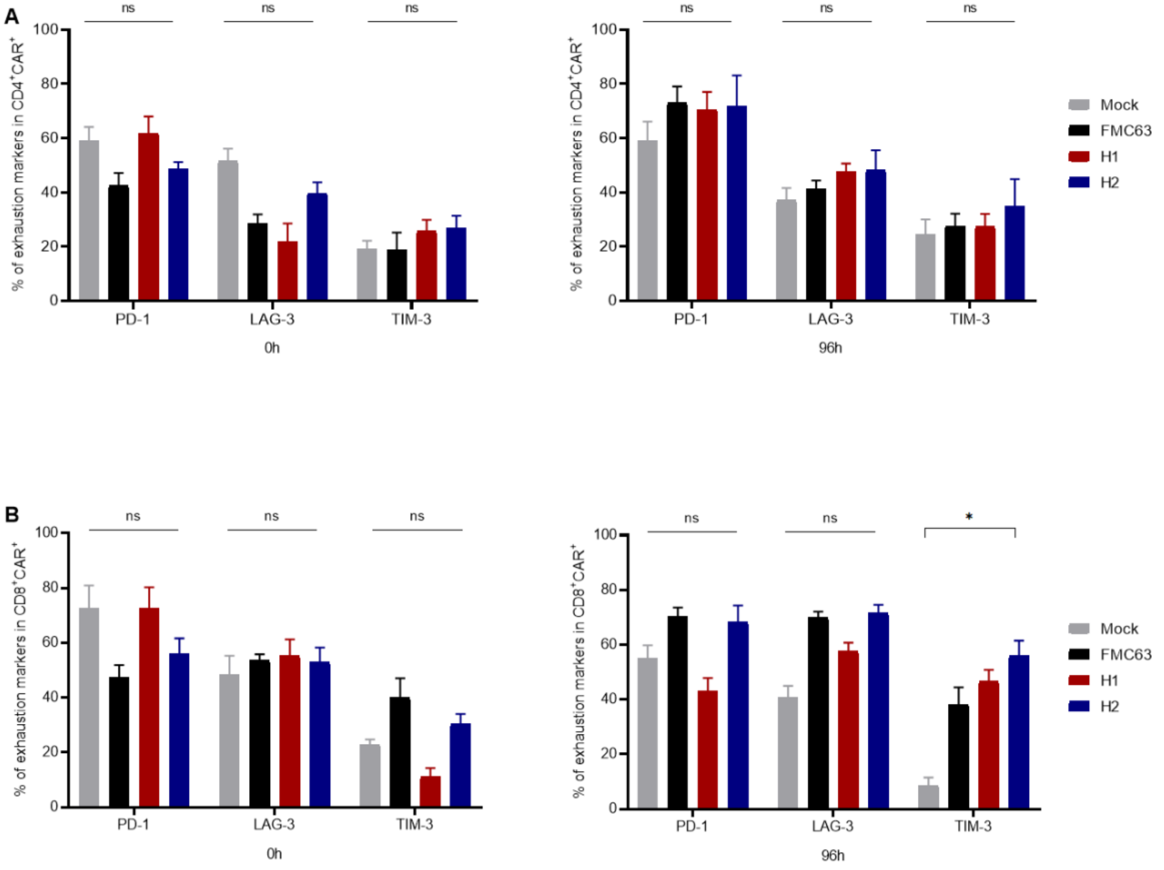

Supplementary Figure S9. Phenotypic exhaustion profile of CAR-T cells following antigen challenge. A. PD1, LAG-3 and TIM3 expression in CD4$^+$ CAR-T cells. B. PD1, LAG-3 and TIM3 expression in CD8$^+$ CAR-T cells. The percentage of positive cells was measured previously (Oh) and after 96h co-culture of FMC63 (black), H1(red) and H2 (blue) CAR$^+$ cells with CD19$^+$ GFP$^+$ Nalm 6 target cells. Mock cells (electroporated, but not transfected, in gray) were also evaluated. Data represent the mean of three independent experiments (n = 3). Statistical significance was assessed via Friedman test (*$p < 0.05$).
